# Immunogenic Cell Death Inducing Fluorinated Mitochondria‐Disrupting Helical Polypeptide Synergizes with PD‐L1 Immune Checkpoint Blockade

**DOI:** 10.1002/advs.202001308

**Published:** 2021-02-01

**Authors:** Seong Dong Jeong, Bo‐Kyeong Jung, Hyo Min Ahn, DaeYong Lee, JongHoon Ha, Ilkoo Noh, Chae‐Ok Yun, Yeu‐Chun Kim

**Affiliations:** ^1^ Department of Chemical and Biomolecular Engineering Korea Advanced Institute of Science and Technology (KAIST) Daejeon 34141 Republic of Korea; ^2^ Department of Bioengineering, College of Engineering Hanyang University Seoul 04763 Republic of Korea; ^3^ GeneMedicine Co., Ltd. Seoul 04763 Republic of Korea; ^4^ Institute of Nano Science and Technology (INST) Hanyang University Seoul 04763 Republic of Korea

**Keywords:** combination cancer immunotherapy, immune checkpoint blockade, immunogenic cell death, mitochondrial membrane destabilization, *α*‐helical polypeptide

## Abstract

Immunogenic cell death (ICD) is distinguished by the release of tumor‐associated antigens (TAAs) and danger‐associated molecular patterns (DAMPs). This cell death has been studied in the field of cancer immunotherapy due to the ability of ICD to induce antitumor immunity. Herein, endoplasmic reticulum (ER) stress‐mediated ICD inducing fluorinated mitochondria‐disrupting helical polypeptides (MDHPs) are reported. The fluorination of the polypeptide provides a high helical structure and potent anticancer ability. This helical polypeptide destabilizes the mitochondrial outer membrane, leading to the overproduction of intracellular reactive oxygen species (ROS) and apoptosis. In addition, this oxidative stress triggers ER stress‐mediated ICD. The in vivo results show that cotreatment of fluorinated MDHP and antiprogrammed death‐ligand 1 antibodies (*α*PD‐L1) significantly regresses tumor growth and prevents metastasis to the lungs by activating the cytotoxic T cell response and alleviating the immunosuppressive tumor microenvironment. These results indicate that fluorinated MDHP synergizes with the immune checkpoint blockade therapy to eliminate established tumors and to elicit antitumor immune responses.

## Graphical abstract

Endoplasmic reticulum (ER) stress‐mediated immunogenic cell death (ICD) inducing fluorinated mitochondria‐disrupting helical polypeptides (MDHPs) are developed. Intracellular oxidative stress by mitochondrial outer membrane destabilization triggers ER stress‐mediated ICD. In addition, combination regimen of fluorinated MDHP and immune checkpoint inhibitor significantly regresses tumor growth and prevents metastasis to the lungs by activating the cytotoxic T cell response.

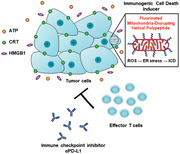

## 1 Introduction

Immune checkpoint blockade has revitalized the field of cancer immunotherapy due to the promising therapeutic effects by clinically inducing a potent antitumor immune response.^[^ ^1^ ^]^ Despite the great success of the immune checkpoint inhibitor (ICI) therapies, ICI monotherapy provokes unsatisfactory long‐term antitumor effects in most patients.^[^ ^2^ ^]^ One of the major pieces of evidence of a low response against ICI therapy is the poorly immunogenic tumor microenvironment, which is the so‐called “cold tumor” resulting from 1) defects in antigen presentation toward T cells,^[^ ^3^ ^]^ 2) absence of T cell activation, 3) lack or minority of activated T cell infiltration in tumor tissues,^[^ ^4^ ^]^ and 4) abundance of immune suppressor cells such as regulatory T cells (Tregs) and myeloid‐derived suppressor cells (MDSCs).^[^ ^5^ ^]^ Therefore, the development of a combination regimen with other therapeutic agents that can convert cold tumors into hot tumors is urgently needed.

Specifically, immunogenic cell death (ICD) has gained much attention for provoking an unusual form of cell death. ICD is a distinctive form of cell demise, which elicits antitumor immune responses by releasing tumor‐associated antigens (TAAs) and danger‐associated molecular patterns (DAMPs).^[^ ^6^, ^7^ ^]^ While cancer cells are undergoing ICD, antigen‐presenting cells (APCs) such as dendritic cells (DCs) and macrophages engulf TAAs and conduct antigen processing and presentation to T cells. During these processes, DAMPs, which are a distinct characteristic of ICD, mediate the activity of DCs to promote adaptive immune responses.^[^ ^8^ ^]^ 1) Secreted adenosine triphosphate (ATP), a “find me” signal, recruits DCs into the tumor site and induces the secretion of IL‐1*β*, a proinflammatory cytokine required for differentiation into IFN‐*γ*‐producing, tumor‐specific cytotoxic T lymphocytes.^[^ ^9^ ^]^ 2) Calreticulin (CRT), acting as an “eat me” signal, is exposed on the cell surface during the early stage of apoptosis and facilitates the phagocytosis of DCs.^[^ ^10^ ^]^ 3) Extracellular release of high‐mobility group box 1 (HMGB1) during postapoptotic cell death leads to DC maturation.^[^ ^11^ ^]^

Thus, recent studies on ICD have been intensively carried out to research the area of cancer immunotherapy.^[^ ^12^ ^]^ To strongly stimulate the release of DAMPs, simultaneous endoplasmic reticulum (ER) stress and reactive oxygen species (ROS) production have a vital role.^[^ ^13^ ^]^ Strategies for impairing ER homeostasis or mitochondria have been established in an effort to exert ER stress and oxidative stress at the same time, resulting in an enhanced immune response against the tumor.^[^ ^6^, ^14^ ^]^ Previously, we designed helical polypeptides that are capable of inducing mitochondria‐dependent apoptosis and targeting the mitochondria.^[^ ^15^, ^16^ ^]^ The helical polypeptides were able to target and destabilize mitochondrial membranes and accelerated oxidative stress, leading to apoptosis. Their mitochondria‐disrupting property is likely to be exploited as an ICD inducer due to the formation of intracellular oxidative conditions.

**Figure 1:**
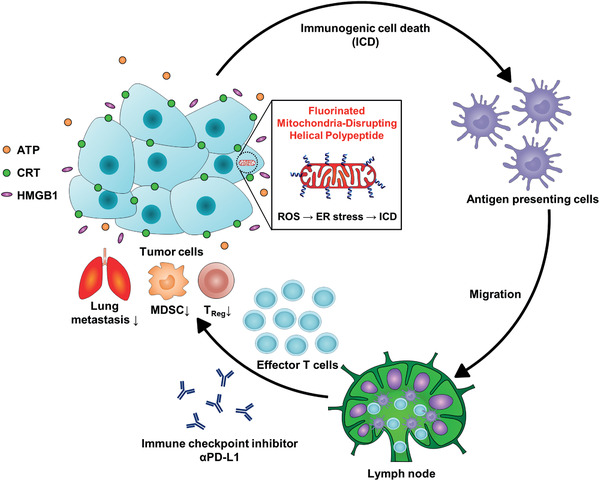
ICD inducing fluorinated MDHPs potentiate immune checkpoint blockade therapy to induce antitumor immune responses. Fluorinated MDHPs destabilize mitochondrial outer membrane and generate intracellular ROS. This oxidative stress elicits ER stress‐mediated ICD. APCs engulf TAAs and migrate to lymph node, followed by differentiating naïve T cells into tumor‐specific effector T cells. ICD inducing fluorinated MDHPs potentiate immune checkpoint blockade therapy, resulting in elimination of established CT26 tumor. In addition, this combination cancer immunotherapy regimen prevents lung metastasis and reduces population of immune suppressor cells such as MDSCs and Tregs.

Herein, we developed fluorinated mitochondria‐disrupting helical polypeptides (MDHPs), for which perfluoroalkyl chains were introduced to the side chains of the polypeptide to ameliorate the bioapplicability and to provide amphipathicity to the polypeptide.^[^ ^17^ ^]^ The fluorinated MDHPs were able to exert ER stress on cells and then release DAMPs, thereby enhancing the antitumor immunity (**Figure** **1**). We hypothesized that the MDHPs were capable of targeting mitochondria and eliciting mitochondrial dysfunction, thereby inducing ER stress‐mediated ICD. Moreover, the appropriate fluorination of the polypeptide showed unique characteristics such as a high helical propensity and robust anticancer activity. The fluorinated MDHP also more strongly triggered ER stress‐mediated ICD and ER stress‐mediated apoptosis than that of the nonfluorinated one. In this study, we demonstrated that fluorinated MDHP‐mediated disruption of the mitochondria considerably inflicted ER stress on the cells and enabled DAMPs to be rapidly released. To overcome the limitations of ICI monotherapy, we evaluated the therapeutic efficacy of the combination of anti‐programmed death‐ligand 1 antibody (*α*PD‐L1) and fluorinated MDHP, which was developed in this study. The coadministration of *α*PD‐L1 and fluorinated MDHP not only highly eliminated the established murine colon adenocarcinoma CT26 tumor but also significantly reduced tumor metastasis to the lungs. These results prove that the ICD‐inducing helical polypeptide significantly enhances both the activation and intratumoral infiltration of CD4^+^ and CD8^+^ T cells and APCs when coadministered with *α*PD‐L1, and thus, the fluorinated MDHP potentiates the therapeutic efficacy of the immune checkpoint blockade by improving the immunosuppressive tumor microenvironment (Figure 1). Therefore, the combination cancer immunotherapy demonstrated a generalizable strategy for eliciting enhanced antitumor immune responses that can eliminate tumors.

## 2 Results and Discussion

### 2.1 Characterization of Fluorinated MDHPs

**Figure 2:**
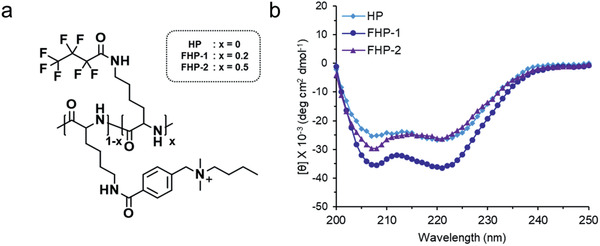
a) Chemical structure of fluorinated MDHPs and b) CD spectra.

Circular dichroism (CD) spectrometry was used to verify the secondary structure of the synthesized polypeptides (**Figure** **2**). We introduced 20 and 50 mol% of perfluoroalkyl chains to the side chains of a polypeptide (HP: 0 mol%; FHP‐1: 20 mol%; FHP‐2: 50 mol%). All the polypeptides adopted an *α*‐helical structure due to the elongation of the charged side chains from the polypeptide backbone.^[^ ^18^ ^]^ In addition, the appropriate fluorination of the polypeptide side chain showed a significant enhancement of the helicity compared to the nonfluorinated and 50 mol% fluorinated polypeptides. This increased helical content is attributed to the augmentation of the hydrophobicity of the polypeptide by introducing perfluoroalkyl chains.

### 2.2 Fluorinated MDHP‐Mediated Mitochondrial Outer Membrane Destabilization

To identify the cellular penetration mechanism of the fluorinated MDHPs, a cellular uptake study was conducted. CT26 cells were pretreated with the endocytic inhibitors chlorpromazine (CPZ), methyl‐*β*‐cyclodextrin (m*β*CD), and 5‐(*N*‐ethyl‐*N*‐isopropyl)amiloride (EIPA) or incubated at 4 °C. After 30 min, the cells were treated with the polypeptides. All the polypeptides showed a reduction of the uptake level at 4 °C and under the EIPA pretreatment conditions, which validates that our polypeptides were internalized into the cells via energy‐dependent endocytosis and macropinocytosis except for direct penetration (Figure S12, Supporting Information). For FHP‐2, the cellular uptake level was slightly decreased by CPZ, indicating clathrin‐mediated endocytosis affects the intracellular uptake of FHP‐2. We next examined endo/lysosomal escape of polypeptides by confocal laser scanning microscopy (CLSM). The green fluorescence of internalized polypeptides was considerably separated from LysoTracker Red, indicating internalized polypeptides effectively escaped endo/lysosome (Figure S13, Supporting Information).

**Figure 3:**
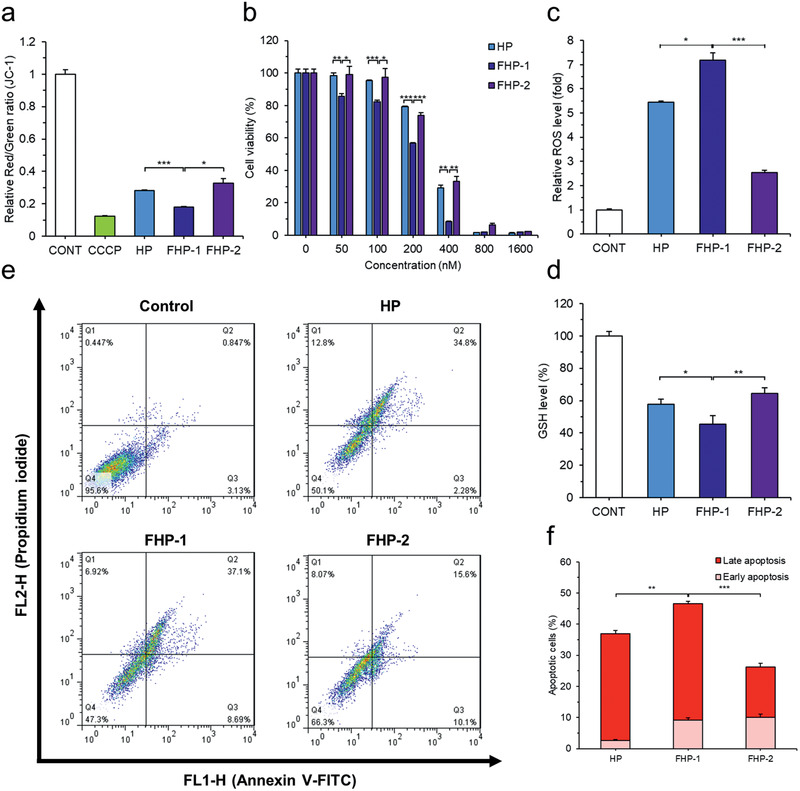
Cytotoxic effect and induction of apoptosis by fluorinated MDHPs. a) Depolarization of mitochondrial membrane potential was confirmed by JC‐1 assay. b) Cytotoxic effect of polypeptides was evaluated by MTT (3‐(4,5‐dimethylthiazol‐2‐yl)‐2,5‐diphenyltetrazolium bromide) assay. Quantification of c) Intracellular relative ROS level and d) GSH level (%) to verify intracellular oxidative condition. e) Cells were stained with Annexin V‐FITC and PI for apoptosis assay and analyzed by flow cytometry. f) Quantification of apoptosis rate. Data are presented as mean ± S.D. (*n* = 3). *P < 0.05, **P < 0.01, ***P < 0.001, unpaired Student's *t*‐test.

The mitochondrial outer membrane disrupting ability of the fluorinated MDHPs was then evaluated. The JC‐1 (5,5′,6,6′‐tetrachloro‐1,1′,3,3′‐tetraethylbenzimidazolycarbocyanine iodide) assay showed the depolarization of the mitochondrial membrane potential in the cell through a change in the ratio of JC‐1 aggregates and monomers (**Figure** **3**a). All the polypeptides elicited a mitochondrial membrane potential loss, suggesting that the internalized polypeptides destabilize the mitochondrial outer membrane resulting in mitochondrial dysfunction. Among them, FHP‐1 caused the most severe damage to mitochondria.

### 2.3 Cytotoxic Effect and Induction of Apoptosis by Exposure to Fluorinated MDHPs

Cell viability was assessed to confirm the potential of the fluorinated MDHPs as an anticancer agent. As shown in Figure 3b, the fluorinated MDHPs exhibited a dose‐dependent cytotoxic effect in the CT26 cells. It was determined that FHP‐1 has the lowest half‐maximal inhibitory concentration (IC_50_) of 181.4 × 10^−9^ m compared to HP and FHP‐2. In addition, to corroborate the anticancer activity of fluorinated MDHP in other cancer cell lines (HCT116 (human colon carcinoma) and murine Lewis lung carcinoma (LLC)), we measured cell viabilities at various concentrations. Similar to the previous result in CT26, FHP‐1 showed the highest cytotoxicity in both HCT116 and LLC cell lines (Figure S14, Supporting Information). FHP‐1 possesses a robust anticancer activity due to its rigidity of the perfluoroalkyl chain and higher helicity.^[^ ^19^ ^]^ Furthermore, it is important to balance the hydrophobicity and cationic charge density in the development of MDHPs.

Mitochondrial dysfunction leads to cellular oxidative stress through increased ROS level and depletion of glutathione (GSH). We evaluated the intracellular ROS and GSH levels using 2′,7′‐Dichlorofluorescin diacetate (DCF‐DA) and Ellman's reagent, respectively (Figure 3c,d). It was observed that FHP‐1 induced a remarkable ROS generation compared to the nontreated control group (7.17‐fold increase, FHP‐1; HP, 5.45‐fold; FHP‐2, 2.54‐fold), which is consistent with the results for the depolarization of the mitochondrial membrane potential. As expected, the polypeptide treatment provoked a significant decrease in the GSH level by ≈45–64%. Taken together, the fluorinated MDHPs effectively exert cellular oxidative stress.

It has been reported that cells undergo characteristic changes during apoptosis, other than necrosis. Various methods, such as cell morphology changes and flow cytometry, have been used to assess apoptosis. First, after the polypeptide treatment, nuclear morphological changes were observed by 4ʹ,6‐diamidino‐2‐phenylindole (DAPI) staining. When the cells were exposed to the polypeptides, nuclear condensation occurred in the apoptotic cells (Figure S15, Supporting Information). In addition, it was confirmed by western blotting that when the polypeptides were treated, the expression level of cleaved caspase 3 increased (Figure S16, Supporting Information). To confirm the apoptosis‐inducing ability of the polypeptides by flow cytometry, cells treated with the fluorinated MDHPs for 24 h were stained with annexin V‐fluorescein isothiocyanate (FITC) and propidium iodide (PI) (Figure 3e,f). It was found that the fluorinated MDHPs have the ability to elicit apoptosis, and the degree of apoptosis in the FHP‐1 group was the highest among all the groups, indicating destabilization of the mitochondrial outer membrane and cellular oxidative stress ultimately result in apoptotic cell death.

### 2.4 ER Stress‐Mediated ICD

**Figure 4:**
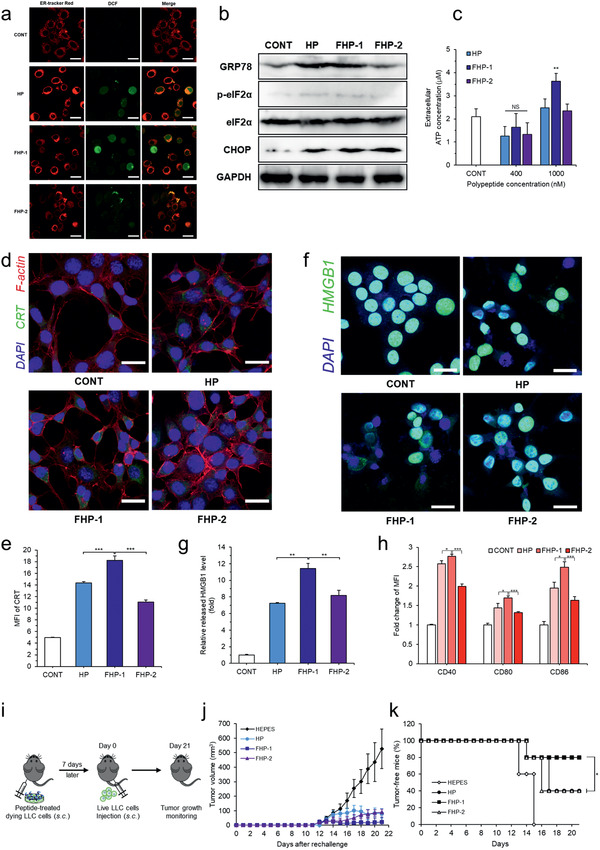
ER stress‐mediated ICD. a) Intracellular ROS and ER were visualized by CLSM. Colocalization of intracellular ROS and ER indicates oxidative stress is applied on ER. b) Western blotting of ER stress related proteins. c) Cells were treated with polypeptides and supernatants were collected to measure extracellular ATP concentration. d) CRT exposure on the cell surface was observed by CLSM after polypeptides treatment and e) mean fluorescence intensity (MFI) of CRT was measured by flow cytometry. f) CLSM of HMGB1 release from the nucleus and g) relative released HMGB1 level measured by ELISA. h) Fold change of MFI of BMDC maturation markers (CD40, CD80, and CD86) analyzed by flow cytometry (gated on CD11c^+^). Data are presented as mean ± S.D. (*n* = 3). i) Treatment schedule for vaccination experiment in LLC mouse model. j) Average tumor growth curves after rechallenge. k) Percentages of tumor‐free mice after rechallenge. Data are presented as mean ± S.D. (*n* = 5). NS: nonsignificant, *P < 0.05, **P < 0.01, ***P < 0.001, unpaired Student's *t*‐test or one‐way ANOVA with Tukey's multiple comparisons test. The scale bar represents 20 µm.

ER stress and overgeneration of ROS are prominent factors related to the induction of ICD. Therefore, we investigated whether the generated ROS are able to stimulate ER. Intracellular ROS was visualized by DCF‐DA, followed by ER‐tracker Red is used to stain the ER. As shown in **Figure** **4**a, we identified that oxidative stress was applied to the ER through the colocalization of ER‐tracker Red and DCF. In addition, expression of ER stress‐related marker proteins, glucose‐regulated protein 78 (GRP78), phosphorylated eukaryotic translation initiation factor 2*α* (p‐eIF2*α*), and C/EBP homologous protein (CHOP), were confirmed by western blotting (Figure 4b; Figure S17, Supporting Information). Cells treated with fluorinated MDHPs showed a higher expression level of GRP78, p‐eIF2*α*, and CHOP than those treated with HP, suggesting the fluorinated MDHPs elicited ROS‐mediated ER stress.

Based on the above results, we evaluated whether the fluorinated MDHPs can serve as an ICD inducer. DAMPs such as the secretion of ATP, CRT exposure, and HMGB1 release are essential to elicit the ICD of cancer cells. A high concentration of fluorinated MDHPs induced a significant secretion of ATP, which recruits DCs into the tumor sites (Figure 4c). The preapoptotic surface exposure of CRT was examined by CLSM and flow cytometry (Figure 4d,e). Fluorinated MDHPs triggered a notable CRT exposure on the cancer cell surface compared to the no‐treatment control. Furthermore, we demonstrated that FHP‐1 treatment in HCT116 and LLC cells increased CRT exposure level by flow cytometry (Figure S18, Supporting Information). Released HMGB1 from the nucleus was visualized with CLSM, and the relative released HMGB1 level was measured by enzyme‐linked immunosorbent assay (ELISA) (Figure 4f,g). We demonstrated that nuclear HMGB1 was released extracellularly by the polypeptide treatment, and FHP‐1 had an 11.4‐fold greater relative released HMGB1 level compared to the control group. Therefore, we validated that the fluorinated MDHPs effectively induced ICD in cancer cells through the identification of ICD hallmarks.

We next studied bone marrow‐derived dendritic cell (BMDC) maturation by CT26 cells where ICD occurred due to the polypeptide treatment. BMDCs were cocultured with the polypeptide pretreated CT26 cells for 24 h and dendritic cell maturation markers such as CD40, CD80, and CD86 were measured by flow cytometry. As shown in Figure 4h, FHP‐1 exhibited the highest upregulation level of CD40, CD80, and CD86 among all the groups, which is consistent with the results of ICD induction. These results indicated that induction of ICD in cancer cells by fluorinated MDHPs promoted dendritic cell maturation.

To further evaluate the effects of ICD by fluorinated MDHP, a vaccination experiment, a gold‐standard approach to detect ICD in vivo,^[^ ^20^ ^]^ was performed in LLC tumor model, which is known to be poorly immunogenic.^[^ ^21^ ^]^ Briefly, LLC cells (1 × 10^6^) were pretreated with fluorinated MDHP or control group, and dying LLC cells were administered subcutaneously 7 days prior to injection of healthy LLC cells (1 × 10^6^) to study the effect of vaccination on tumorigenesis (Figure 4i). The mice vaccinated with FHP‐1 treated LLC cells had the highest vaccination efficacy as demonstrated by the lowest percentage of tumor establishment rate (HEPES (4‐(2‐Hydroxyethyl)piperazine‐1‐ethanesulfonic acid), 100%; HP, 60%; FHP‐1, 20%; and FHP‐2, 60%) and the slowest tumor growth rate (Figure 4j,k; Figure S19, Supporting Information). These data indicate that FHP‐1 administration could efficiently induce ICD in poorly immunogenic tumors.

### 2.5 Tumor Inhibition Study of a Fluorinated MDHP and *α*PD‐L1 Combination Regimen

**Figure 5:**
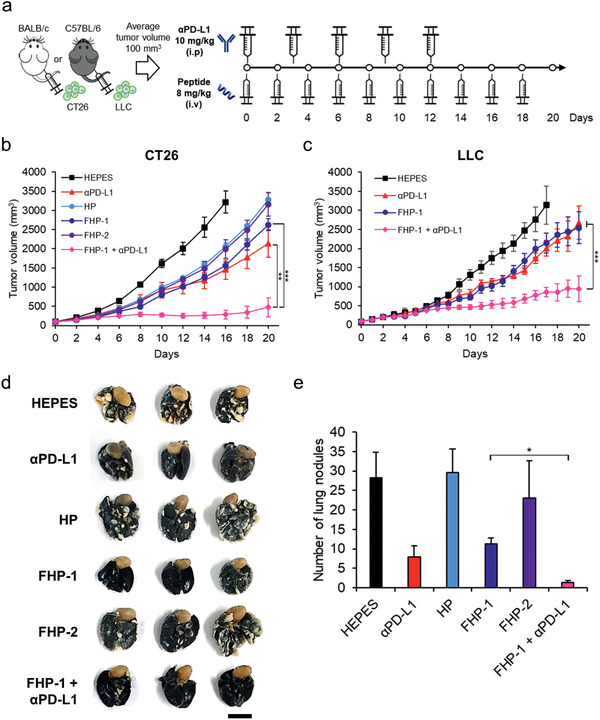
Tumor inhibition study of fluorinated MDHP with *α*PD‐L1. a) Treatment schedule. CT26 or LLC cells were injected subcutaneously to BALB/c mice or C57BL/6 mice and when the average volume of established tumor reached 100 mm^3^, the tumors were injected with helical peptides (HP, FHP‐1, or FHP‐2; 8 mg kg^−1^) intravenously on 10 times every other day and/or *α*PD‐L1 (10 mg kg^−1^) intraperitoneally on every three days for 5 times. b,c) Average tumor growth curves of CT26 or LLC bearing mice after treatment with polypeptide and/or *α*PD‐L1. The tumor volume was measured every other day until the end of the study. d) Representative images of lung metastatic tumor nodules. Lungs were collected on day 35 and stained with India ink. The scale bar represents 1 cm. e) The number of lung metastatic tumor nodules in mice. Data are presented as mean ± S.D. (*n* = 6). *P < 0.05, **P < 0.01, ***P < 0.001, one‐way ANOVA with Tukey's multiple comparisons test.

To assess the antitumor efficacy of the *α*PD‐L1 and FHP‐1 combination regimen, CT26 murine colorectal tumor‐bearing mice were treated with *α*PD‐L1 (10 mg kg^−1^), ICD inducing peptides (HP, FHP‐1 or FHP‐2; 8 mg kg^−1^), or FHP‐1 (8 mg kg^−1^) plus *α*PD‐L1 (10 mg kg^−1^), along with HEPES as a negative control (**Figure** **5**a). As shown in Figure 5b and Figure S20 (Supporting Information), the HEPES‐treated CT26 tumors grew rapidly and aggressively with an average of 3000 mm^3^ by day 16. In contrast, a monotherapy, which was either *α*PD‐L1, HP, FHP‐1, or FHP‐2 alone, used to treat the colorectal tumor‐bearing mice significantly inhibited the tumor growth, showing a 54.8%, 36.0%, 51.6%, and 38.2% smaller average tumor volume, respectively, compared to the HEPES controls on day 16. FHP‐1 showed the best antitumor effect among the three kinds of helical peptides because of the longest in vivo circulation and high tumor accumulation (Figure S21, Supporting Information). In addition, mice coadministered with FHP‐1, which is the most potent ICD inducing peptide among the three, and *α*PD‐L1 showed the most effective antitumor efficacy, resulting in a 1.8‐ or 1.7‐fold higher antitumor effect, respectively, compared to the FHP‐1 (*P* < 0.001) or *α*PD‐L1 monotherapy (*P* < 0.01) on day 16. Further, 42.9% of the mice in the FHP‐1 plus *α*PD‐L1 group showed a complete response, whereas none of the 6 tumors in the monotherapy groups showed complete regression. Importantly, mice coadministered with FHP‐1 and *α*PD‐L1 resulted in a 6‐ or 8.69‐fold lower number of metastatic nodules compared to each monotherapy, suggesting the efficient prevention of metastasis to the lungs (Figure 5d,e). In addition to CT26 tumor, the antitumor effect of the FHP‐1 and *α*PD‐L1 combination therapy was also evaluated in poorly immunogenic LLC tumor model. Same treatment plan as those utilized in CT26 tumor model was used for administration of *α*PD‐L1 and/or FHP‐1 in LLC tumor model (Figure 5a). As shown in Figure 5c, single treatment groups induced a lower level of tumor growth inhibitory effect in the poorly immunogenic LLC tumor model than those observed in CT26. In detail, tumor growth inhibition in comparison to HEPES‐treated group on day 16 of treatment for different monotherapy groups; *α*PD‐L1, 54.77% (CT26), and 36.25% (LLC); FHP‐1, 51.61% (CT26), or 31.80% (LLC). Despite these trends, coadministration of *α*PD‐L1 and FHP‐1 still exerted significantly more potent antitumor effect than either monotherapies in poorly immunogenic LLC model (*P* < 0.001). Together, these results suggest that FHP‐1, in combination with *α*PD‐L1, is a therapeutic regimen that can induce potent antitumor effects in tumor models regardless of their immunogenicity level.

### 2.6 Histological and Immunohistochemical Analysis

To further investigate the mechanism of the FHP‐1 and/or *α*PD‐L1‐induced therapeutic effect, tumor tissues were extracted at 2 days after the last FHP‐1 treatment from each group and then assessed by histological and immunological analysis. As shown in **Figure** **6**a, hematoxylin and eosin (H&E) staining revealed a large area of proliferating tumor cells in the HEPES‐treated tumor tissues, whereas a moderate or extensive necrosis region was detected in the tumor tissues with the *α*PD‐L1‐ and/or FHP‐1 treatment. In line with the H&E staining, the TUNEL assay showed that the largest proportion of cancer cells had undergone apoptosis due to the combination therapy. The CRT, GRP78, p‐eIF2*α*, and CHOP expression levels revealed that the PD‐L1 blockade induced a mild ER stress, whereas the FHP‐1 peptides effectively stimulated the ER stress in both the single and coadministration with *α*PD‐L1 (Figure 6a; Figures S22 and S23, Supporting Information). These results suggest that the FHP‐1 peptide is an effective ER stress inducer, leading to ER stress‐mediated apoptosis, and these phenomena are further enhanced by coadministration with a PD‐L1 blockade.

Figure 6Histological and immunohistochemical analysis. a) Tumor tissues were collected at 2 days after the last treatment and stained with hematoxylin and eosin (H & E; top row), terminal deoxynucleotidyl transferase dUTP nick end labeling (TUNEL; middle row), and CRT‐specific Ab (bottom row). Original magnification: ×100 for H&E and TUNEL images or ×400 for CRT staining. The scale bar represents 10 µm for H&E and TUNEL images and 2 µm for CRT images. The quantitative data represented as mean ± S.D. (*n* = 3); ** *P* < 0.01, ***P < 0.001. b) IFN‐*γ* ELISpot assay. Splenocytes were harvested from HEPES‐, *α*PD‐L1‐, FHP‐1‐, or FHP‐1 plus *α*PD‐L1‐treated mice on 2 days following the last treatment, and coincubated with preirradiated CT26 cells for 16 h with various effector to target (ET) ratio. Then, the IFN‐*γ* ELISpot assay was carried out. Data represented as mean ± S.D. (*n* = 3). c) FACS analysis for MDSC and Treg. Splenocytes harvested from mice received each treatment were stained with CD11b‐ and Gr‐1‐specific Ab for MDSC analysis and stained with CD4‐, CD25‐, and FoxP3‐specific Ab. Data represented as mean ± S.D. (*n* = 3); * *P* < 0.05, **P < 0.01, ***P < 0.001. d–l) FACS analysis of tumor infiltrating immune cells in LLC mouse model. d) CD4^+^ T cells, e) CD8^+^ T cells, f) DCs, g) M1 macrophages, h) M2 macrophages, i) NK cells, j) MDSCs, k) IFN‐*γ* ^+^CD4^+^ T cells, and l) IFN‐*γ* ^+^CD8^+^ T cells. (Group 1: HEPES, Group 2: *α*PD‐L1, Group 3: FHP‐1, Group 4: FHP‐1+*α*PD‐L1.) Data are presented as mean ± S.D. (*n* = 3). *P < 0.05, **P < 0.01, ***P < 0.001, one‐way ANOVA with Tukey's multiple comparisons test.

**Figure:**
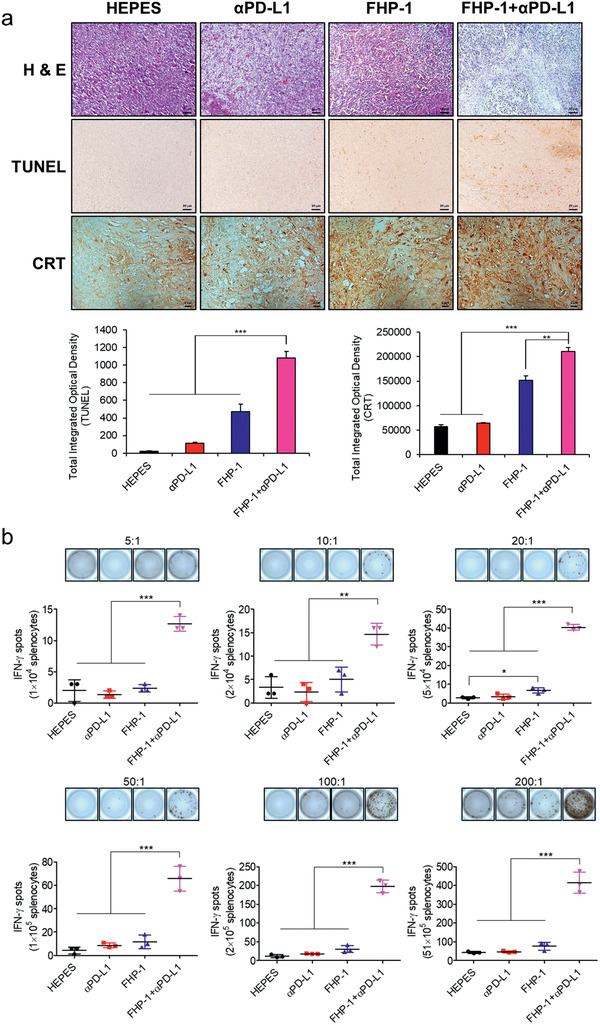

**Figure:**
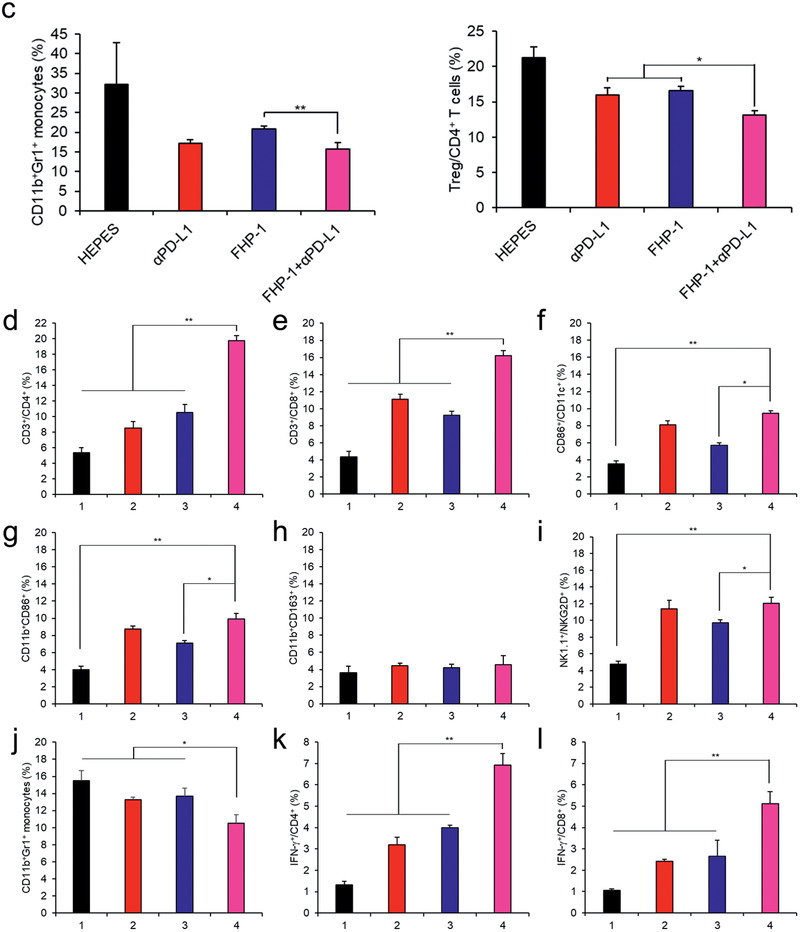

### 2.7 Activation of the Immune Response by the Combination of the Fluorinated MDHPs and PD‐L1 Blockade

To explore the mechanism from the immunological aspects of the enhanced antitumor efficacy, the immune cell population in tumor tissue and spleen was investigated. Coadministration of FHP‐1 and *α*PD‐L1 led to the highest level of CD4^+^ and CD8^+^ T cell infiltration into both CT26 and LLC tumor tissues (Figure 6d,e; Figure S24, Supporting Information). In addition, the number of activated DCs, NK cells, and M1 macrophages in tumor tissues were also significantly higher in combination therapy group than those observed in other treatment groups (*P* < 0.05 or *P* < 0.01; Figure 6f,g,i; Figures S24 and S25, Supporting Information). Moreover, the frequency of T cells secreting IFN‐*γ* via stimulation with cancer cells by at least 2.9‐ up to 5.6‐fold compared to the FHP‐1 alone treatment (*P* < 0.001; Figure 6b). Activated CD4^+^ and CD8^+^ T cells were also detected at the highest level in tumor tissues that were treated with FHP‐1 and *α*PD‐L1 combination therapy (Figure 6k,l; *P* < 0.01 versus other groups). On the other hand, the number of M2 macrophages following in combination therapy group was similar to those observed in HEPES‐treated group (Figure 6h). Still, the number of immunosuppressive MDSC was significantly decreased in tumor tissues following coadministration of FHP‐1 and *α*PD‐L1 in respect to other treatments (*P* < 0.05; Figure 6j), which is in agreement with significant reduction in Treg and MDSC counts in spleen upon administration of combination therapy in CT26 tumor‐bearing mice (Figure 6c). In addition, immune cell analysis in CT26 mouse model showed similar trends to the above results (Figure S26, Supporting Information). Overall, these results suggest that the ER stress‐mediated apoptosis induced by FHP‐1 increases the formation of tumor antigens to activate DCs, a representative APC, and to trigger activation and intratumoral infiltration of CD4^+^ and CD8^+^ T cells while attenuating the number of immunosuppressive immune cells in tumor or a lymphoid organ in both immunogenic (CT26) and poorly immunogenic (LLC) tumor tissues. These phenomena were further promoted when combined with PD‐L1 blockade. Thus, a combination therapeutic regimen of FHP‐1 and *α*PD‐L1 could improve the therapeutic efficacy by defeating the negative phenomena seen in patients with a low response to immune checkpoint blockade monotherapy.

To confirm whether antitumor effect of combination of FHP‐1 and *α*PD‐L1 depends on antitumor immune responses, we carried out a tumor inhibition study using immunocompromised BALB/c nude mice. As shown in Figure S27 (Supporting Information), there was no significant difference in the tumor inhibition effect between combination therapy and each monotherapy. The change of body weight in all groups was negligible. Therefore, these results demonstrate that enhanced therapeutic efficacy of combination therapy in immunocompetent mice relies on antitumor immunity.

### 2.8 Toxicity Profile

**Figure 7:**
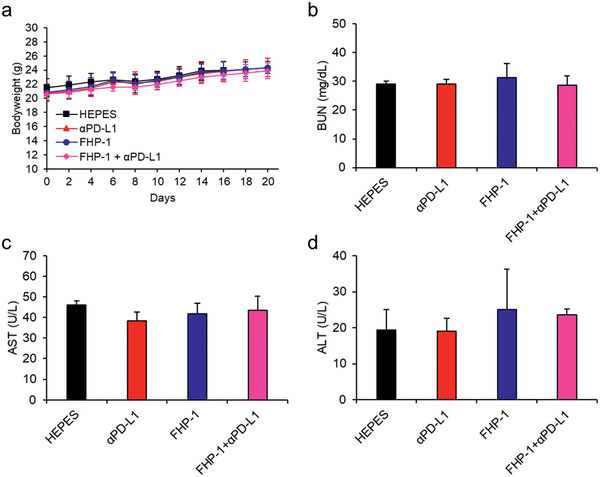
Toxicological profile. a) Body weight measurement. CT26‐bearing mice were treated with the same regimen of antitumor efficacy study and body weight was measured every other day. Data represented as mean ± S.D. (*n* = 6). b–d) CT26 tumor‐bearing mice were treated with *α*PD‐L1, FHP‐1, and FHP‐1 plus *α*PD‐L1 along with HEPES as a control. Blood was collected on day 2 after the last FHP‐1 treatment, and BUN for kidney toxicity and AST and ALT for liver toxicity were determined for each mouse. Data represented as mean ± S.D. (*n* = 3).

To determine the potential toxicity of the *α*PD‐L1 and FHP‐1 alone or in the combination treatment, the bodyweight changes in mice were measured every other day, and serum chemistry was carried out for kidney and liver toxicity (**Figure** **7**; Table S1, Supporting Information). None of the treatments significantly affected the bodyweight loss; moreover, the levels of blood urea nitrogen (BUN), bilirubin, aspartate aminotransferase (AST), and alanine aminotransferase (ALT) in all individuals were in the normal range. Taken together, these results demonstrate that the *α*PD‐L1 and FHP‐1 combination therapeutic strategy is safe.

## 3 Conclusion

Induction of mitochondrial dysfunction is crucial to trigger apoptosis for effective cancer therapy.^[^ ^22^ ^]^ Previous studies showed that MDHPs destabilize the mitochondrial outer membrane and provoke apoptosis by ROS overgeneration.^[^ ^16^ ^]^ Although these MDHPs have been proven to induce apoptosis and effectively inhibit tumor growth, the subsequent antitumor immunity has not been studied. To activate the immune system against cancer, cancer cells undergo a special form of cell death, releasing TAAs and various DAMPs, known as ICD.^[^ ^6^, ^7^ ^]^

In this study, we developed ER stress‐mediated ICD inducing helical polypeptide that synergizes with *α*PD‐L1 to induce antitumor immunity. For an enhanced bioapplicability and potent anticancer activity, we introduced perfluoroalkyl chains to the side chains of the helical polypeptides. These fluorinated MDHPs led to mitochondrial depolarization by destabilization of the mitochondrial outer membrane and exerted severe oxidative stress through augmentation of intracellular ROS and a decrease in the GSH level. Moreover, we assessed the apoptosis‐inducing ability of the fluorinated MDHPs and their cytotoxic effect in CT26 cells. FHP‐1 had the highest apoptosis rate and the lowest IC_50_ value. When considering the apoptosis‐inducing ability of the fluorinated MDHPs, we hypothesized that excessive intracellular ROS induce ER stress, thereby eliciting ICD, and sensitization of the tumor cells by the ICD‐inducing helical polypeptides enhances the efficacy of the immune checkpoint inhibitor. We demonstrated that intracellular ROS generated by the treatment of fluorinated MDHPs stimulated the ER, and the expression of ER stress‐related marker proteins, GRP78 and CHOP, was confirmed by western blotting. To verify the ICD‐inducing ability of the fluorinated MDHPs, we assessed the characteristic biomarkers of ICD. The exposure of CRT and the release of ATP and HMGB1 were observed in cancer cells treated with the helical polypeptides. CT26 or LLC tumor‐bearing mice were used to demonstrate the tumor inhibition effect of the helical polypeptides and their synergistic effect with immune checkpoint blockade therapy. Combination cancer immunotherapy of the fluorinated MDHP and *α*PD‐L1 remarkably inhibited tumor growth and metastasis to the lung. This therapeutic regimen activated immune responses against a tumor and decreased the population of immune suppressor cells such as MDSCs and Tregs. Our results suggest that fluorinated MDHPs as an ICD inducer unequivocally have a potent synergistic effect with immune checkpoint blockade therapy for effective combination cancer immunotherapy.

## 4 Experimental Section

##### Circular Dichroism (CD) Measurements

CD measurements were performed on a J‐815 spectropolarimeter 150‐L type (JASCO, Japan) to identify the secondary structure of the polypeptides. Polypeptide samples were prepared in deionized (DI) water at a concentration of 0.5 mg mL^−1^ at pH = 7. CD spectra were measured using a quartz cell with a path length of 0.2 mm in the range of 200–250 nm at room temperature (RT).

##### Mitochondrial Membrane Potential Assay

CT26 cells were cultured in 12‐well plates at 15 × 10^4^ cells per well for 24 h and treated with 200 × 10^−9^ m of HP, FHP‐1, and FHP‐2 in fresh serum‐free DMEM (Dulbecco's Modified Eagle Medium). After the treatment, cells were washed with PBS (Phosphate Buffered Saline) and harvested for JC‐1 (5,5′,6,6′‐tetrachloro‐1,1′,3,3′‐tetraethylbenzimidazolycarbocyanine iodide) (10 µg mL^−1^, Invitrogen Co., Carlsbad, CA, USA) staining at 37 °C for 15 min. Additionally, 50 × 10^−6^ m of CCCP was added simultaneously with JC‐1 as a positive control. Subsequently, the cells were collected and diluted with the medium for flow cytometry analysis (FACS Calibur, BD Biosciences, CA, USA).

##### Cell Viability Test

To measure the cytotoxicity of the HP, FHP‐1, and FHP‐2, the MTT (3‐(4,5‐dimethylthiazol‐2‐yl)‐2,5‐diphenyltetrazolium bromide) assay was conducted. CT26, HCT116, and LLC cells were seeded on 96‐well plates at 1 × 10^4^ cells per well and incubated for 24 h. The medium was replaced with various concentrations of HP, FHP‐1, and FHP‐2 in serum‐free DMEM. After a 24 h incubation, 20 µL of MTT (5 mg mL^−1^ in PBS) were added to each well. Following a further 3 h incubation, the medium was removed, and 150 µL of DMSO were added to dissolve the formazan crystal. The absorbance at 570 nm was measured using a microplate reader (Multiskan GO, Thermo Scientific, Waltham, USA).

##### Intracellular ROS levels

CT26 cells were seeded on 12‐well plates at 15 × 10^4^ cells per well. After incubation for 24 h, fresh serum‐free DMEM that included 200 × 10^−9^ m of HP, FHP‐1, and FHP‐2 was added to each well and incubated for 24 h. Subsequently, the cells were washed with PBS and stained with 10 × 10^−6^ m of DCF‐DA at 37 °C for 30 min. After staining, the harvested cells were analyzed using flow cytometry (FACS Calibur, BD Biosciences).

##### Intracellular GSH levels

CT26 cells were cultured in 12‐well plates at 15 × 10^4^ cells per well for 24 h, followed by the incubation of 200 × 10^−9^ m of HP, FHP‐1, and FHP‐2 in fresh serum‐free DMEM. Cells were rinsed with PBS prior to adding 500 µL of RIPA buffer and incubated at 4 °C for 30 min. After further incubation, cell lysates were centrifuged, and 20 µL of Ellman's reagent (0.75 × 10^−3^ m DTNB) were added to the supernatant. The absorbance was measured at 405 nm using a microplate reader (Multiskan GO, Thermo Scientific).

##### Apoptosis Assay

CT26 cells were seeded on 12‐well plates at a density of 15 × 10^4^ cells per well and cultured for 24 h. The cells were treated with 200 × 10^−9^ m of HP, FHP‐1, and FHP‐2 in fresh serum‐free DMEM. After a 24 h incubation, the medium was removed, and the cells were rinsed with PBS. The cells were collected and stained with Annexin V‐FITC and propidium iodide (Invitrogen Co., Carlsbad, CA, USA). The stained cells were analyzed by flow cytometry (FACS Calibur, BD Biosciences).

##### Visualization of ROS and ER

4.5 × 10^4^ CT26 cells were seeded in µ‐Slide 8 well (Ibidi, USA) for 24 h and then treated with 100 × 10^−9^ m of HP, FHP‐1, and FHP‐2. After 24 h, the cells were washed with PBS prior to incubation with 10 × 10^−6^ m of DCF‐DA at 37 °C for 30 min. Following washing with PBS, the cells were stained with ER‐Tracker Red (500 × 10^−9^ m, Invitrogen Co.) at 37 °C for 30 min. The stained cells were washed with PBS containing Pluronic F‐127 (0.1%) and visualized with confocal laser scanning microscopy (LSM 800 META, ZEISS, Germany).

##### Western Blotting Analysis for ER‐Stress Related Marker Proteins

50 × 10^4^ CT26 cells were seeded in 6‐well plates for 24 h and then treated with 200 × 10^−9^ m of HP, FHP‐1, and FHP‐2. After a 24 h incubation, the cells were washed with PBS and lysed with RIPA buffer with protease inhibitor cocktail (Sigma) and phosphatase inhibitor cocktail (Sigma). Extracted proteins were quantified by the BCA kit. Proteins were loaded into each well of a sodium dodecyl sulfate‐polyacrylamide gel (SDS‐PAGE), and electrophoresis was performed. Subsequently, the proteins were transferred to a polyvinylidene fluoride (PVDF) membrane. The PVDF membrane was blocked with 5 wt% skim milk in tris‐buffer saline with 0.05% Tween 20 (TBST) solution for 1 h, followed by incubation with primary antibodies (Cleaved Caspase‐3 antirabbit polyclonal (Cell Signaling Technology, Beverly, MA, USA), glyceraldehyde 3‐phosphate dehydrogenase (GAPDH) antirabbit polyclonal (Santa Cruz Bio Technology, USA), GRP78 antirabbit monoclonal (Cell Signaling Technology), p‐eIF2*α* antirabbit polyclonal (Cell Signaling Technology), eIF2*α* antirabbit polyclonal (Cell Signaling Technology), and CHOP antirabbit polyclonal (Abcam)) at 4 °C overnight. Then, the membrane was washed with TBST, and horseradish peroxidase (HRP)‐conjugated antirabbit (Abcam) was added as a secondary antibody and incubated at 4 °C overnight. Blots were developed by enhanced chemiluminescent (ECL) reagent (GE healthcare, USA).

##### Extracellular ATP Concentration

1 × 10^4^ CT26 cells cultured in 96‐well plates were treated with serum‐free DMEM containing 400 and 1000 × 10^−9^ m of HP, FHP‐1, and FHP‐2 for 24 h. Collected supernatants were used to measure the extracellular ATP concentration with the ATP Colorimetric/Fluorometric Assay Kit (BioVision, USA) according to the manufacturer's instructions.

##### In Vitro CRT Expression

50 × 10^4^ CT26, HCT116, and LLC cells were seeded in 6‐well plates and then cultured for 24 h. The medium was replaced with 300 × 10^−9^ m of HP, FHP‐1, and FHP‐2. Following a 24 h incubation, the cells were washed with PBS and harvested. The harvested cells were incubated with a rabbit anti‐CRT antibody (Cell Signaling Technology) at 4 °C for 30 min. Then, the cells were washed with PBST and stained with Alexa Fluor 488‐conjugated goat antirabbit IgG (Abcam) at 4 °C for 30 min. After washing with PBST, the stained cells were analyzed by flow cytometry (FACS Calibur, BD Biosciences).

For CLSM imaging of the CRT expression, 8 × 10^4^ CT26 cells were seeded on coverslips in a 24‐well plate and incubated for 24 h. Cells were treated with HP, FHP‐1, and FHP‐2 for 24 h and washed with PBS. The cells were fixed with 4% paraformaldehyde at 37 °C for 10 min, followed by blocking with 1% BSA in PBST at RT for 1 h. Then, the cells were incubated with a rabbit anti‐CRT antibody (Cell Signaling Technology) at 4 °C overnight. After the overnight incubation, the cells were washed with PBST, and Alexa Fluor 488‐conjugated goat antirabbit IgG (Abcam) was added to each well and incubated at RT for 2 h. To stain the cytoskeleton, the cells were incubated with Alexa Fluor 647 Phalloidin (Cell Signaling Technology) at RT for 30 min. After washing with PBST, the cells were stained with DAPI at RT for 10 min and mounted onto slides. The cells were visualized with confocal laser scanning microscopy (LSM 800 META, ZEISS).

##### Measurement of Released HMGB1

1 × 10^4^ CT26 cells cultured in 96‐well plates were treated with serum‐free DMEM in the presence of 200 × 10^−9^ m of HP, FHP‐1, and FHP‐2 for 24 h. Then, supernatants were collected by centrifugation at 1500 rpm for 10 min at 4 °C, and 20 µL of each supernatant were diluted in coating buffer (carbonate–bicarbonate buffer, Sigma). Next, 100 µL of diluted supernatant were coated onto 96‐well plates and incubated at 4 °C overnight. Subsequently, each well was blocked with 5% skim milk (Sigma) in PBS‐Tween 20 (PBST, 0.05% v/v, Sigma) at RT for 2 h. After washing with PBST, rabbit anti‐HMGB1 antibody (Abcam) diluted in PBST with 2.5% skim milk was added to the plates and incubated at 4 °C overnight. Then, the plates were washed with PBST and horseradish peroxidase (HRP)‐conjugated goat antirabbit IgG (Abcam) was added to the plates and incubated at RT for 2 h. After the final washing, the plates were developed with *o*‐phenylenediamine dihydrochloride (OPD) at RT for 30 min. To stop the reaction, 3 n HCl solution was added to each well, and the optical density (OD) was measured at an absorbance of 492 nm using a microplate reader (Multiskan GO, Thermo Scientific).

For confocal laser scanning microscopy (CLSM) imaging of HMGB1, 8 × 10^4^ CT26 cells were seeded on coverslips in a 24‐well plate and cultured for 24 h. The cells were treated with HP, FHP‐1, and FHP‐2 for 24 h and washed with PBS. Subsequently, the cells were fixed with 4% paraformaldehyde at 37 °C for 10 min. After fixation, the cells were blocked and permeabilized with 1% bovine serum albumin (BSA) and 0.1% Triton X‐100 in PBST at RT for 1 h. Rabbit anti‐HMGB1 antibody (Abcam) solution was added to each well and incubated at 4 °C overnight. Then, the cells were washed with PBST, and Alexa Fluor 488‐conjugated goat anti‐rabbit IgG (Abcam) was added to each well and incubated at RT for 2 h. After the final washing, the cells were stained with DAPI at RT for 10 min and mounted onto slides. Cells were visualized with confocal laser scanning microscopy (LSM 800 META, ZEISS).

##### In Vitro Bone Marrow‐Derived Dendritic Cell (BMDC) Maturation

BMDCs were generated from the bone marrow of 10 week old BALB/c mice. 7 × 10^5^ BMDCs were seeded in 12‐well plates and cocultured with HP, FHP‐1, and FHP‐2 pretreated CT26 cells for 24 h. After incubation, the cells were collected and blocked with antimouse CD16/32 antibody (Biolegend, CA, USA) for 10 min. Subsequently, the cells were stained with anti‐CD11c antibody, anti‐CD40 antibody, anti‐CD80 antibody, and anti‐CD86 antibody (Biolegend) for 30 min. The stained cells were analyzed by flow cytometry (FACS Fortessa, BD Biosciences).

##### Animal Experiments

All the experimental procedures involving animal studies were performed in accordance with the NIH Guideline for the Care and Use of Laboratory Animals. These procedures were approved by the Institutional Animal Care and Use Committee of Hanyang University.

##### Vaccination Experiment

The mice were injected with 1 × 10^6^ of dying LLC cells, which had been pretreated with 500 × 10^−9^ m of HP, FHP‐1, or FHP‐2 for 24 h, on the left flank. A week later, 1 × 10^6^ healthy LLC cells were injected into the right flank. The tumor incidence rate and tumor growth curve were monitored until 21 days after the second tumor cell inoculation.

##### Antitumor Efficacy in a Mouse Tumor Model

Murine colon carcinoma tumor was subcutaneously established by injecting 5 × 10^5^ cells of CT26 in 100 µL of Hank's balanced salt solution (HBSS; Gibco‐BRL, Grand Island, NY) into the right flanks of 6‐week‐old male BALB/c mice (Daehan Biolink Co., Ltd, Chungbuk, Korea). Murine LLC tumors were established by subcutaneously injecting 1 × 10^6^ LLC cells in 100 µL of Hank's balanced salt solution (HBSS; Gibco‐BRL) into the right flanks of 6‐week‐old male C57BL/6 mice (Daehan Biolink Co., Ltd). NCI‐H460 cells (5 × 10^6^ cells in 100 µL of Hank's balanced salt solution (HBSS; Gibco‐BRL)) were subcutaneously injected into 6‐week‐old male BALB/c nude mice (Orient Bio Inc., Seongnam, Korea). When the average tumor volume reached 100 mm^3^, HEPES, *α*PD‐L1, HP, FHP‐1, FHP‐2, or FHP‐1 plus *α*PD‐L1 were administered to the tumor‐bearing mice. All helical peptides (HP, FHP‐1, and FHP‐2) were injected intravenously (8 mg kg^−1^) every other day for a total of 10 times, and *α*PD‐L1 was injected intraperitoneally (10 mg kg^−1^) every three days for a total of 5 times. The first day of treatment was set as day 0. Tumor volume was determined by measuring the length (*L*) and width (*W*) of each tumor with a caliper every other day. The tumor volume was calculated using the following equation: tumor volume = 0.523 *L*(*W*)^2^. Bodyweight was also measured every other day.

##### Lung Metastasis

To assess the metastasis in the lungs, the same treatment schedule, as described above, was used. Twenty‐five days after the last treatment, the CT26 tumor‐bearing mice were sacrificed, and 2 mL of 10% of India Ink solution (Hardy Diagnostics, Santa Maria, CA) were injected into the lungs via the trachea. After the stained lungs were extracted from the mice, these were rinsed with Fekete's solution (70% ethanol, 4% formaldehyde, glacial acetic acid; 20:2:1)^[^ ^23^ ^]^ and were placed in fresh Fekete's solution overnight. Then, the stained lungs were stored in 70% ethanol before further analysis. Photographs were obtained, and the visible foci were counted.

##### Histology and Immunohistochemistry

For the histologic analysis, tumor tissues were collected from mice at 2 days post last FHP‐1 injection, fixed in 10% formalin, embedded in paraffin, and sectioned at a thickness of 5 µm. Representative sections were stained with H&E solution. For immunohistochemical staining, sectioned tumor tissues were blocked with Protein Block Serum Free Ready To Use (Dako, Glostrup, Denmark) for 2 h and incubated with CHOP antibody (9C8; Abcam), CRT antibody (FMC75; Abcam), CD11c (HL3; BD Pharmingen), or CD83 (HB15e; BD Pharmingen) as a primary antibody. After washing, the sections were incubated with the Dako Envision Kit (Dako as a secondary antibody, and then counterstained with Meyer's hematoxylin (Sigma). A TUNEL assay was performed using the ApopTag Peroxidase In Situ Apoptosis Detection Kit (Millipore, Billerica, MA, USA) according to the manufacturer's instructions. To identify the lymphocytes in the tumor tissues, the tumor tissues were frozen in OCT compound (Sakura Finetec, Torrance, CA, USA) and cut into 10 µm thick sections. The cryosections were blocked with protein block serum free ready to use (Dako) and incubated with anti‐CD86 antibody (GL1; BD Biosciences), anti‐CD4 antibody (H129.19; BD Biosciences), or anti‐CD8 Antibody (53‐6.7; BD Biosciences) as the primary antibody and biotin‐conjugated goat anti‐rat IgG (BD PharMingen, San Diego, CA) as the secondary antibody. Then, the tumor tissue slices were treated with streptavidin‐HRP (BD Pharmingen). Diaminobenzidine/hydrogen peroxidase (Dako) was used as the chromogen substrate. All slides were counterstained with Meyer's hematoxylin.

##### Interferon (IFN)‐*γ* ELISpot Assay

CT26 tumor‐bearing mice were administered with HEPES, *α*PD‐L1, FHP‐1, or FHP‐1 plus *α*PD‐L1. FHP‐1 was injected intravenously (8 mg kg^−1^) every other day for a total of 6 times, and *α*PD‐L1 was injected intraperitoneally (10 mg kg^−1^) every three days for a total of 4 times. Spleens were collected from the mice at 2 days post 6th FHP‐1 injection, and splenocytes were prepared as described previously.^[^ ^24^ ^]^ An IFN‐*γ* ELISpot assay was then carried out following the manufacturer's instructions. The positive spots, representing IFN‐*γ*‐producing cells, were counted using an ELISpot plate reader (AID iSpot FluoroSpot; Autoimmun Diagnostika GmbH, Strassberg, Germany) and analyzed with the AID EliSpot software Version 7.0 (Autoimmun Diagnostika GmbH).

##### FACS Analysis

CT26 or LLC tumor‐bearing mice were administered with HEPES, *α*PD‐L1, FHP‐1, or FHP‐1 plus *α*PD‐L1. FHP‐1 was injected intravenously (8 mg kg^−1^) every other day for a total of 6 times, and *α*PD‐L1 Abs were injected intraperitoneally (10 mg kg^−1^) every three days for a total of 4 times. Spleens and tumor tissues were collected from mice at 2 days post 6th FHP‐1 injection, and splenocytes and tumor infiltrating immune cells were prepared as previously reported.^[^ ^24^ ^]^ All cells were pretreated with saturating anti‐CD16/CD32 Ab (Biolegend, San Diego, CA) in staining buffer (2% FBS, 0.02% sodium azide in PBS) to block cellular Fc receptors before staining with primary Abs. Then, primary Abs corresponding to each immune cell were treated for 30 min at 4 °C, washed with PBS three times and fixed with 1% paraformaldehyde. The primary Abs used are specified in Table S2 (Supporting Information). All samples were analyzed on a BD FACS Calibur flow cytometer (Becton Dickinson, Cockeysville, MD).

##### Serum Chemistry

Serum levels (U L^−1^ or mg dL^−1^) of AST, ALT, BUN, and total bilirubin were measured by the Hitachi 7600 DDP modular chemistry analyzer (Seegene Inc., Seoul, Korea).

##### Statistical Analysis

All data were expressed as the mean ± standard deviation (S.D.). All Statistical comparisons were performed by unpaired Student's *t*‐test or one‐way ANOVA with Tukey's multiple comparisons test using GraphPad Prism (version 4.0 for Windows, GraphPad Software, San Diego CA). The criterion for statistical significance was taken as *P* < 0.05.

## Conflict of Interest

H.M.A. is employee of GeneMedicine. C.‐O.Y. is the CEO in GeneMedicine. The remaining authors declare no conflict of interest.

## Supporting information

Supporting Information

## References

[1] a) G. Q. Phan , J. C. Yang , R. M. Sherry , P. Hwu , S. L. Topalian , D. J. Schwartzentruber , N. P. Restifo , L. R. Haworth , C. A. Seipp , L. J. Freezer , Proc. Natl. Acad. Sci. USA 2003, 100, 8372; 12826605 10.1073/pnas.1533209100 PMC166236

[2] a) S. Champiat , L. Dercle , S. Ammari , C. Massard , A. Hollebecque , S. Postel‐Vinay , N. Chaput , A. Eggermont , A. Marabelle , J.‐C. Soria , Clin. Cancer Res. 2017, 23, 1920; 27827313 10.1158/1078-0432.CCR-16-1741

[3] M. L. Broz , M. Binnewies , B. Boldajipour , A. E. Nelson , J. L. Pollack , D. J. Erle , A. Barczak , M. D. Rosenblum , A. Daud , D. L. Barber , Cancer Cell 2014, 26, 638. 25446897 10.1016/j.ccell.2014.09.007 PMC4254577

[4] E. Peranzoni , J. Lemoine , L. Vimeux , V. Feuillet , S. Barrin , C. Kantari‐Mimoun , N. Bercovici , M. Guérin , J. Biton , H. Ouakrim , Proc. Natl. Acad. Sci. USA 2018, 115, E4041. 29632196 10.1073/pnas.1720948115 PMC5924916

[5] R. Weber , V. Fleming , X. Hu , V. Nagibin , C. Groth , P. Altevogt , J. Utikal , V. Umansky , Front. Immunol. 2018, 9, 1310. 29942309 10.3389/fimmu.2018.01310 PMC6004385

[6] D. V. Krysko , A. D. Garg , A. Kaczmarek , O. Krysko , P. Agostinis , P. Vandenabeele , Nat. Rev. Cancer 2012, 12, 860. 23151605 10.1038/nrc3380

[7] D. R. Green , T. Ferguson , L. Zitvogel , G. Kroemer , Nat. Rev. Immunol. 2009, 9, 353. 19365408 10.1038/nri2545 PMC2818721

[8] a) L. Galluzzi , A. Buqué , O. Kepp , L. Zitvogel , G. Kroemer , Nat. Rev. Immunol. 2017, 17, 97; 27748397 10.1038/nri.2016.107

[9] F. Ghiringhelli , L. Apetoh , A. Tesniere , L. Aymeric , Y. Ma , C. Ortiz , K. Vermaelen , T. Panaretakis , G. Mignot , E. Ullrich , Nat. Med. 2009, 15, 1170. 19767732 10.1038/nm.2028

[10] M. Obeid , A. Tesniere , F. Ghiringhelli , G. M. Fimia , L. Apetoh , J.‐L. Perfettini , M. Castedo , G. Mignot , T. Panaretakis , N. Casares , Nat. Med. 2007, 13, 54. 17187072 10.1038/nm1523

[11] C. W. Bell , W. Jiang , C. F. Reich III , D. S. Pisetsky , Am. J. Physiol. 2006, 291, C1318. 10.1152/ajpcell.00616.2005 16855214

[12] a) X. Duan , C. Chan , W. Lin , Angew. Chem., Int. Ed. 2019, 58, 670; 10.1002/anie.201804882 PMC7837455 30016571

[13] a) O. Kepp , L. Menger , E. Vacchelli , C. Locher , S. Adjemian , T. Yamazaki , I. Martins , A. Q. Sukkurwala , M. Michaud , L. Senovilla , Cytokine Growth Factor Rev. 2013, 24, 311; 23787159 10.1016/j.cytogfr.2013.05.001

[14] D. T. Rutkowski , R. J. Kaufman , Trends Cell Biol. 2004, 14, 20. 14729177 10.1016/j.tcb.2003.11.001

[15] D. Lee , I. Noh , J. Yoo , N. S. Rejinold , Y.‐C. Kim , Acta Biomater. 2017, 57, 187. 28528116 10.1016/j.actbio.2017.05.040

[16] a) D. Lee , S.‐H. Lee , Y. Na , I. Noh , J. Ha , J. Yoo , H. B. Bang , J. H. Park , K. J. Jeong , C.‐O. Yun , J. Controlled Release 2017, 264, 24; 10.1016/j.jconrel.2017.08.001 28778477

[17] a) M. P. Krafft , Adv. Drug Delivery Rev. 2001, 47, 209; 10.1016/s0169-409x(01)00107-7 11311993

[18] H. Lu , J. Wang , Y. Bai , J. W. Lang , S. Liu , Y. Lin , J. Cheng , Nat. Commun. 2011, 2, 206. 21343924 10.1038/ncomms1209

[19] Y.‐B. Huang , L.‐Y. He , H.‐Y. Jiang , Y.‐X. Chen , Int. J. Mol. Sci. 2012, 13, 6849. 22837667 10.3390/ijms13066849 PMC3397499

[20] O. Kepp , L. Senovilla , I. Vitale , E. Vacchelli , S. Adjemian , P. Agostinis , L. Apetoh , F. Aranda , V. Barnaba , N. Bloy , Oncoimmunology 2014, 3, e955691.25941621

[21] M. G. Lechner , S. S. Karimi , K. Barry‐Holson , T. E. Angell , K. A. Murphy , C. H. Church , J. R. Ohlfest , P. Hu , A. L. Epstein , J. Immunother. 2013, 36, 477. 24145359 10.1097/01.cji.0000436722.46675.4a PMC3910494

[22] a) S. W. Tait , D. R. Green , Nat. Rev. Mol. Cell Biol. 2010, 11, 621; 20683470 10.1038/nrm2952

[23] F. M. Yakes , J. Chen , J. Tan , K. Yamaguchi , Y. Shi , P. Yu , F. Qian , F. Chu , F. Bentzien , B. Cancilla , J. Orf , A. You , A. D. Laird , S. Engst , L. Lee , J. Lesch , Y. C. Chou , A. H. Joly , Mol. Cancer Ther. 2011, 10, 2298. 21926191 10.1158/1535-7163.MCT-11-0264

[24] I. K. Choi , Y. Li , E. Oh , J. Kim , C. O. Yun , PLoS One 2013, 8, e67512. 23844018 10.1371/journal.pone.0067512 PMC3701076

