# Supplementary material for: Immunogenic Cell Death Inducing Fluorinated Mitochondria‐Disrupting Helical Polypeptide Synergizes with PD‐L1 Immune Checkpoint Blockade
Source: Adv Sci (Weinh). 2021 Feb 1;8(7):2001308. doi: 10.1002/advs.202001308 (PMC8025002; doi:10.1002/advs.202001308)
Supplement: Supplementary file 1 — Supporting Information [file ADVS-8-2001308-s001.pdf]

WILEY-VCH

© 2020 Wiley-VCH GmbH

ADVANCED SCIENCE
Open Access

Supporting Information

for *Adv. Sci.*, DOI: 10.1002/advs.202001308

Immunogenic Cell Death Inducing Fluorinated Mitochondria-Disrupting Helical Polypeptides Synergizes with PD-L1 Immune Checkpoint Blockade

Seong Dong Jeong, Bo-Kyeong Jung, Hyo Min Ahn, DaeYong Lee, JongHoon Ha, Ilkoo Noh, Chae-Ok Yun, and Yeu-Chun Kim**

Supporting Information

Immunogenic Cell Death Inducing Fluorinated Mitochondria-Disrupting Helical Polypeptides Synergizes with PD-L1 Immune Checkpoint Blockade

Seong Dong Jeong, Bo-Kyeong Jung, Hyo Min Ahn, DaeYong Lee, JongHoon Ha, Ilkoo Noh, Chae-Ok Yun, and Yeu-Chun Kim**

S. D. Jeong, Dr. D. Y. Lee, J. H. Ha, Dr. I. Noh, Prof. Y.-C. Kim
Department of Chemical and Biomolecular Engineering
Korea Advanced Institute of Science and Technology (KAIST)
Daejeon 34141, Republic of Korea
E-mail: dohnanyi@kaist.ac.kr

B.-K. Jung, Dr. H. M. Ahn, Prof. C.-O. Yun
Department of Bioengineering
College of Engineering
Hanyang University
Seoul 04763, Republic of Korea
E-mail: chaeok@hanyang.ac.kr

Prof. C.-O. Yun
Institute of Nano Science and Technology (INST)
Hanyang University
Seoul 04763, Republic of Korea

Dr. H. M. Ahn, Prof. C.-O. Yun
GeneMedicine Co., Ltd.
Seoul 04763, Republic of Korea

Materials

$CuCO_3/Cu(OH)_2$, tetrahydrofuran, *N*,*N*-Dimethylformamide, hexamethyldisilazane, acetonitrile, trifluoroacetic acid, 33 wt% Hydrogen bromide solution in acetic acid, methanol, and triethylamine were purchased from Sigma-Aldrich (St. Louis, MO, USA). L-lysine hydrochloride, 4-(chloromethyl)benzoyl chloride, triphosgene, N^{ε}-Carbobenzoxy-L-lysine, α-Pinene, diisopropylethylamine, sodium iodide, *N*-Butyldimethylamine, heptafluorobutyric anhydride, and fluorescein 5-Isothiocyanate were purchased from Tokyo Chemical Industry Co., Ltd.

Synthesis of (N^{ε}-(4-(chloromethyl)benzoyl)-L-lysine)$_2$·Cu(II)

$CuCO_3/Cu(OH)_2$ (3.63 g, 16.424 mmol) was added into 80 mL of an aqueous solution of L-lysine hydrochloride (3 g, 16.424 mmol). The solution was refluxed for 3 h and filtered to remove any unreacted $CuCO_3$. The filtered solution was cooled to 0 ℃, and $NaHCO_3$ (5.52 g, 65.7 mmol) was added. 4-(chloromethyl)benzoyl chloride (6.21 g, 32.85 mmol) dissolved in tetrahydrofuran (THF) was slowly added into the filtered solution under stirring. After 30 min, a precipitate started to form, and the reaction was conducted overnight at room temperature (RT). The precipitated product was filtered and washed with THF and deionized (DI) water to remove any unreacted 4-(chloromethyl)benzoyl chloride and (L-lysine)$_2$·Cu(II). Finally, the product was obtained as a violet solid powder after lyophilization (Yield : 80%).

Synthesis of N^{ε}-(4-(chloromethyl)benzoyl)-L-lysine based *N*-carboxyanhydride (CMB-L-Lys-NCA)

In a glove box, [N^{ε}-(4-(chloromethyl)benzoyl)-L-lysine]$_2$·Cu(II) (4.34 g, 6.585 mmol) was dissolved in THF and triphosgene (1.95 g, 6.585 mmol) was added. The reaction mixture was refluxed, and the color of the mixture turned brown. During the reaction, the precipitation of $CuCl_2$ occurred. After 3 h, the reaction mixture was cooled to 0 ℃, and it was poured into a separatory funnel with ethyl acetate (100 mL). Subsequently, this mixture was washed with cold saturated EDTA-2Na dihydrate/$NaHCO_3/H_2O$ solution three times. The separated organic layer was dried over $MgSO_4$ and concentrated under vacuum to approximately 1/3 of its initial volume. The concentrated organic layer was poured into excessive n-hexane for recrystallization. The precipitated product was filtered and kept in a vacuum to remove n-hexane. A yellowish solid powder was obtained (Yield : 51%).

Synthesis of N^{ε}-Carbobenzoxy-L-lysine based *N*-carboxyanhydride (CBZ-L-Lys-NCA)

In a glove box, N^{ε}-Carbobenzoxy-L-lysine (1.5 g, 5.35 mmol) was dissolved in THF. Then, α-Pinene (1.7 mL, 10.7 mmol) and triphosgene (0.635 g, 2.14 mmol) were added. The reaction proceeded under continuous stirring at 45 ℃ for 3 h. The reaction mixture was

precipitated with excessive n-hexane. The precipitated product was filtered and kept in a vacuum to remove the n-hexane. A white solid powder was obtained (Yield : 85%).

Synthesis of poly(N^{ε}-(4-(chloromethyl)benzoyl)-L-lysine)-*r*-poly(N^{ε}-Carbobenzoxy-L-lysine) (PCBC)

In a glove box, CMB-L-Lys-NCA and CBZ-L-Lys-NCA at different molar ratios (160:40, or 100:100) were dissolved in anhydrous *N*,*N*-Dimethylformamide (DMF) (5 mL). Subsequently, Hexamethyldisilazane (HMDS) (M/I = 200) and *N*,*N*-Diisopropylethylamine (DIPEA) (100 μl) were added. The reaction proceeded under continuous stirring at RT for 48 h. The polymer was precipitated with DI water and collected by filtration. The product was obtained by lyophilization (Yield : PCBC-1 : 90%, PCBC-2 : 92%).

Synthesis of poly(N^{ε}-(4-(chloromethyl)benzoyl)-L-lysine) (PCB)

In a glove box, CMB-L-Lys-NCA was dissolved in anhydrous DMF (5 mL). Subsequently, Hexamethyldisilazane (HMDS) (M/I = 200) and DIPEA (100 μl) were added. The reaction proceeded under continuous stirring at RT for 48 h. The polymer was precipitated with DI water and collected by filtration. The product was obtained by lyophilization (Yield : 89%).

Synthesis of poly(N^{ε}-(*N*-(4-benzoyl)-*N*,*N*-dimethylbutan-1-aminium)-L-lysine)-*r*-poly(N^{ε}-Carbobenzoxy-L-lysine) (PDAC)

PCBC was dissolved in DMF (10 mL), followed by the addition of sodium iodide (3 equivalents of chloro groups) dissolved in acetonitrile (10 mL) and *N*-Butyldimethylamine (3 equivalents of chloro groups). The reaction was conducted at 80 °C for 48 h under continuous stirring. The resulting product was dialyzed against DI water for 48 h in a dialysis bag with a cutoff molecular weight of 3.5 kDa. The product was obtained by lyophilization (Yield : PDAC-1 : 77%, PDAC-2 : 91%).

Synthesis of poly(N^{ε}-(*N*-(4-benzoyl)-*N*,*N*-dimethylbutan-1-aminium)-L-lysine) (HP)

HP was synthesized in the same manner as above (Yield : 40%).

Acidolysis of CBZ groups

PDAC was dissolved in trifluoroacetic acid (TFA) (6 mL). After complete dissolution of the polypeptide, 33 wt% Hydrogen bromide solution in acetic acid (4 equivalents of CBZ groups) was added. The reaction was conducted at RT for 1 h, and the resulting product was poured into excessive ethyl ether. The precipitated polypeptide was filtered and dried under vacuum (Yield : PDA-1 : 93%, PDA-2 : 98%).

Fluorination of the polypeptides

CBZ deprotected polypeptides were dissolved in methanol (5 mL). Heptafluorobutyric anhydride (5 equivalents of deprotected amine groups) and triethylamine (6 equivalents of deprotected amine groups) were added into the above solution under continuous stirring. The reaction was conducted at RT for 24 h. The polypeptides were purified against 1 M NaCl aqueous solution for 1 day and DI water for 1 day in a dialysis bag with a cutoff molecular weight of 3.5 kDa. The polypeptides were obtained by lyophilization as a white solid powder (Yield : FHP-1 : 85%, FHP-2 : 83%).

Fluorescein 5-Isothiocyanate (FITC) conjugation

In a glove box, CMB-L-Lys-NCA and CBZ-L-Lys-NCA at different molar ratios (196:4, 160:40, 100:100) were dissolved in anhydrous *N*,*N*-Dimethylformamide (DMF) (5 mL). Subsequently, Hexamethyldisilazane (HMDS) (M/I = 200) was added. The reaction proceeded under continuous stirring at RT for 48 h. The polymer was precipitated with DI water and collected by filtration. The product was obtained by lyophilization. The following procedures are the same as those mentioned above. FITC (4 equivalents of CBZ deprotected polypeptide) was added to the CBZ deprotected polypeptides dissolved in methanol. The reaction was conducted at RT for 12 h, followed by fluorination except for the 196:4 polypeptide.

IRDye 800CW conjugation

CBZ deprotected polypeptides were dissolved in PBS (5 mL), and subsequently, IRDye 800CW NHS ester (5 mg mL^{-1} in DMSO, LI-COR, USA) (1 equivalent of CBZ deprotected

polypeptides) was added to the solution. The reaction was conducted at RT for 24 h and dialyzed against DI water for 2 days in a dialysis bag with a molecular cutoff weight of 3.5 kDa. IRDye 800CW conjugated polypeptides were obtained by lyophilization.

Characterization

The molecular weights of the synthesized polypeptides were determined by gel permeation chromatography (GPC) with a Younglin YL9100 HPLC system (Korea). 0.01 M LiBr DMF solution was used as a mobile phase at 35 ℃ with a 1 mL min^{-1} flow rate. The repeating unit of PCB, PCBC-1, and PCBC-2 was approximately 200. The synthesized polypeptides were analyzed by ^1H NMR spectroscopy (Agilent 400MHz 54mm NMR DD2, Agilent Technologies, USA). Deuterated DMSO (DMSO-d_6) and Deuterium oxide (D_2O) were used as the NMR solvents.

Cellular uptake mechanism

15×10^4 CT26 cells were seeded in 12-well plates and incubated for 24 h. After the incubation, the cells were pretreated with various endocytosis inhibitors including chlorpromazine (30×10^{-6} M), methyl-β-cyclodextrin (10×10^{-3} M), and 5-(N-Ethyl-N-isopropyl)amiloride (100×10^{-6} M) at 37 ℃ for 30 min. Subsequently, the medium was replaced with 100×10^{-9} M of FITC conjugated polypeptides in serum-free DMEM prior to further incubation at 37 ℃ and 4 ℃ for 4 h. Then, cells were washed with PBS containing heparin (40 U mL^{-1}) three times to remove any remaining FITC conjugated polypeptides. After washing, the cells were collected and fixed with 4% paraformaldehyde for flow cytometry analysis (FACS Calibur, BD Biosciences, CA, USA).

To investigate endo/lysosomal escape of polypeptide, CT26 cells were seeded on coverslips in a 24-well plate at 8×10^4 cells per well for 24 h and stained with LysoTracker Red (100×10^{-9} M, Invitrogen Co.) at 37 ℃ for 30 min. The stained cells were washed with PBS containing Pluronic F-127 (0.1%) and 200×10^{-9} M of FITC conjugated polypeptides

were treated at 37 ℃ for 3 h. After the treatment, the cells were washed with PBS containing heparin (40 U mL^{-1}) and 0.1% trypan blue was added for 2 min to quench extracellular fluorescence. Then, the cells were washed with PBS and fixed with 4% paraformaldehyde at 37 ℃ for 10 min. After fixation, the cells were stained with DAPI at RT for 10 min and mounted onto slides. Stained cells were visualized with confocal laser scanning microscopy (LSM 800 META, ZEISS, Germany) to observe endo/lysosomal escape of polypeptides.

Nuclear condensation

8×10^4 CT26 cells seeded on coverslips in a 24-well plate were treated with 200×10^{-9} M of HP, FHP-1, and FHP-2 for 24 h. Then, the cells were fixed with 4% paraformaldehyde at 37 ℃ for 10 min. After fixation, the cells were stained with DAPI at RT for 10 min and mounted onto slides. Stained cells were visualized with confocal laser scanning microscopy (LSM 800 META, ZEISS, Germany) to observe nuclear morphological changes.

Biodistribution

The biodistribution studies of αPD-L1, HP, FHP-1, FHP-2, or FHP-1 plus αPD-L1 both in vivo and ex vivo were performed on male BALB/c mice (Daehan Biolink) using an in-vivo imaging system (IVIS) (Xenogen Corp, Alameda, CA). IRdye 800CW conjugated-helical peptides (0.8 mg mL^{-1}) were injected intravenously at 8 mg kg^{-1} (n=3) with HEPES as a negative control (n=3). After 1, 3, 6, 12, 24, and 48 h, mice whole body was imaged with IVIS. Then, the mice were sacrificed, and the organs were isolated (liver, heart, kidney, spleen, lung, stomach, and tumor) at 48 h. The harvested organs were imaged with IVIS. Fluorescent images were obtained from the anesthetized mice using the IVIS imaging system.

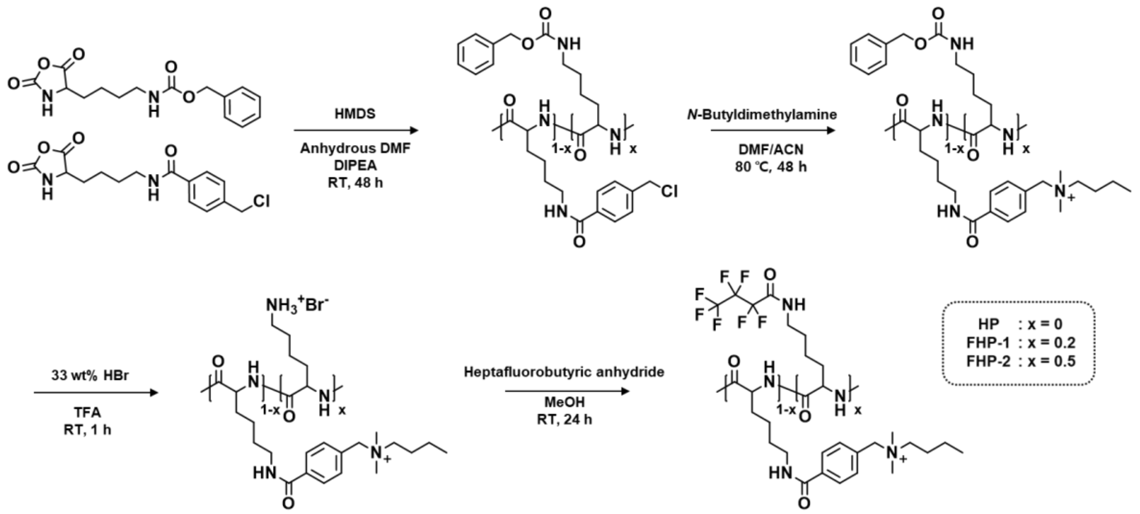

Figure S1. Synthesis scheme of the fluorinated mitochondria-disrupting helical polypeptides.

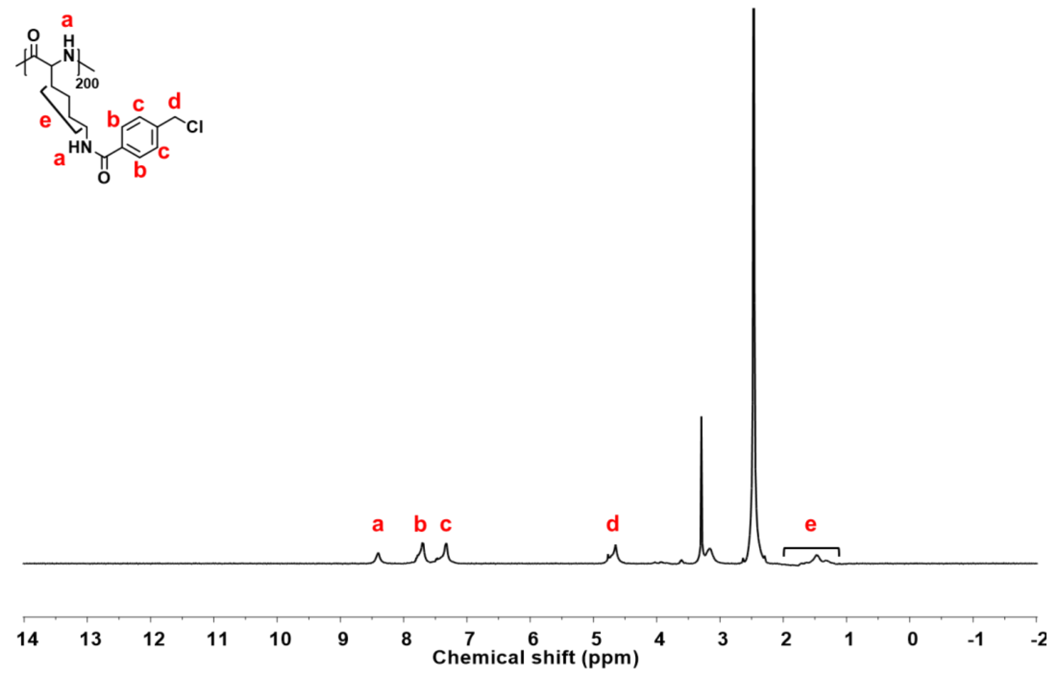

Figure S2. ^{1}H NMR of PCB in DMSO-d_6

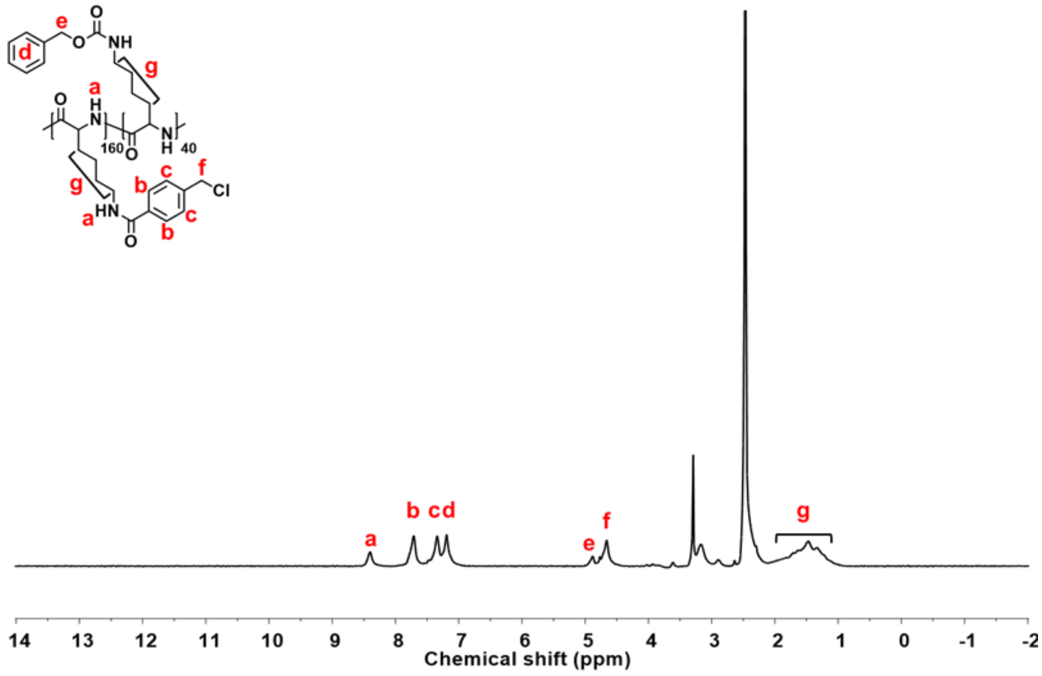

Figure S3. ^{1}H NMR of PCBC-1 in DMSO-d_6

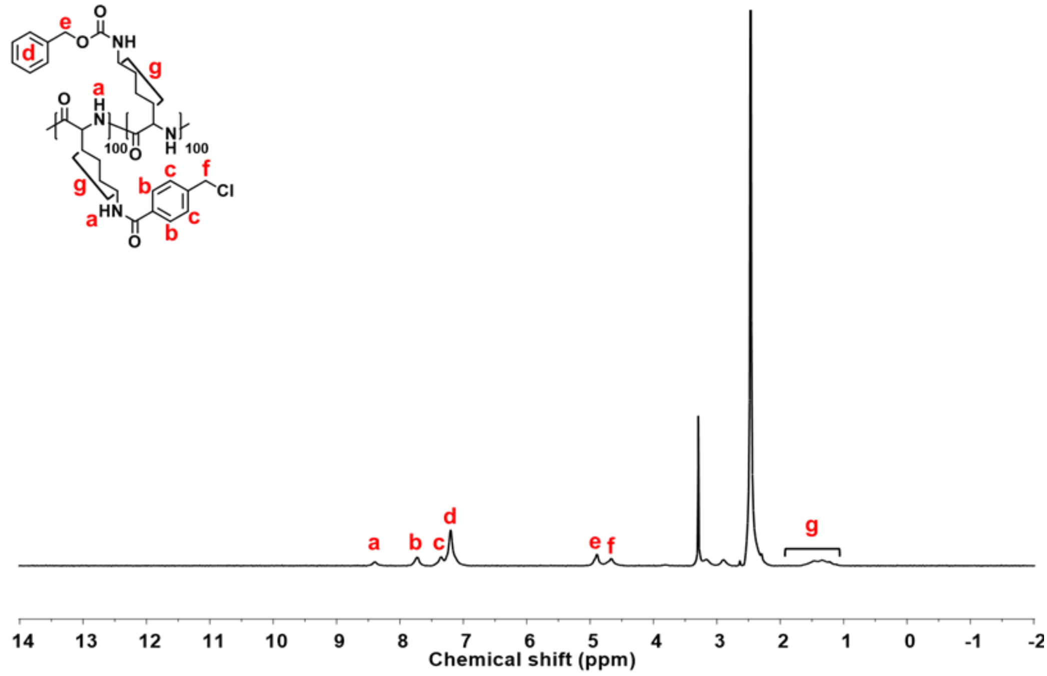

Figure S4. ^{1}H NMR of PCBC-2 in DMSO-d_6

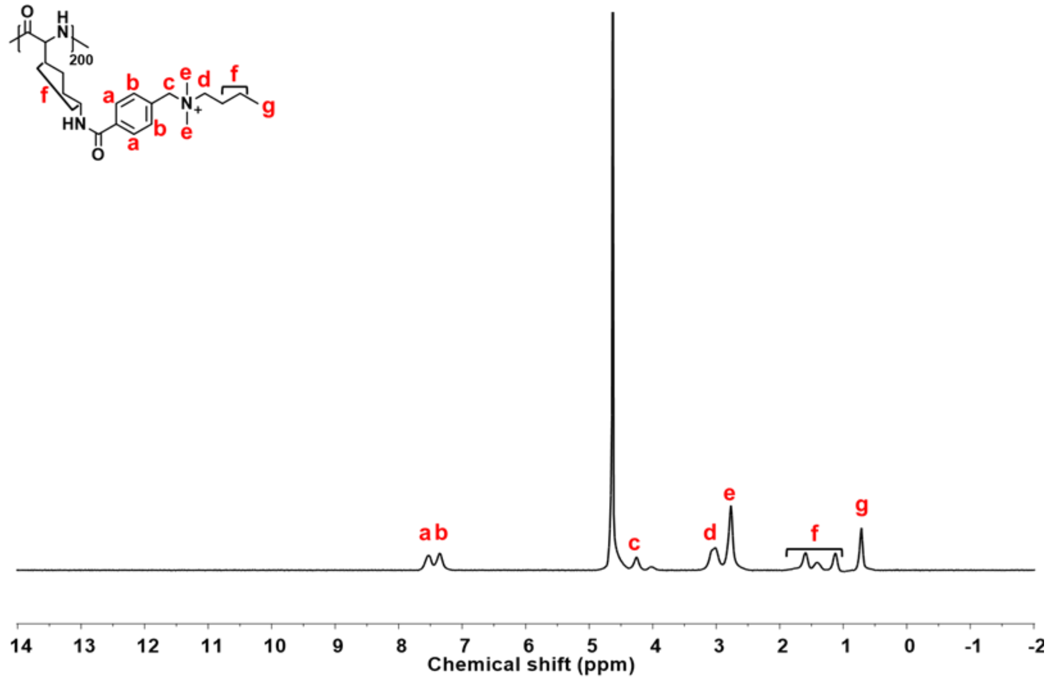

Figure S5. ^{1}H NMR of HP in D_2O

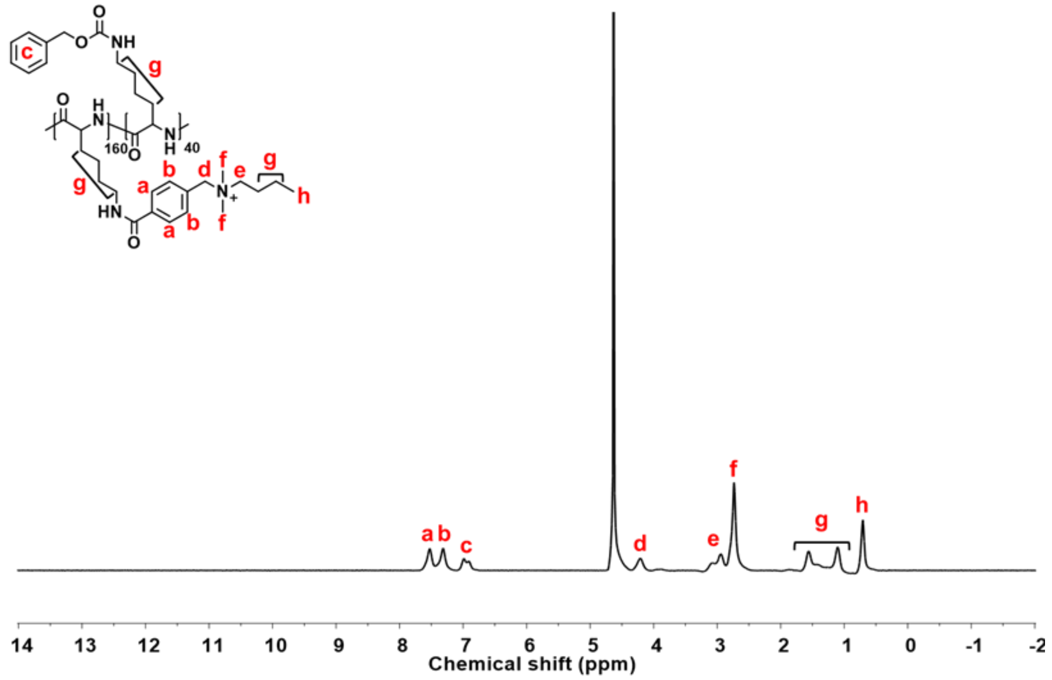

Figure S6. ^{1}H NMR of PDAC-1 in D_2O

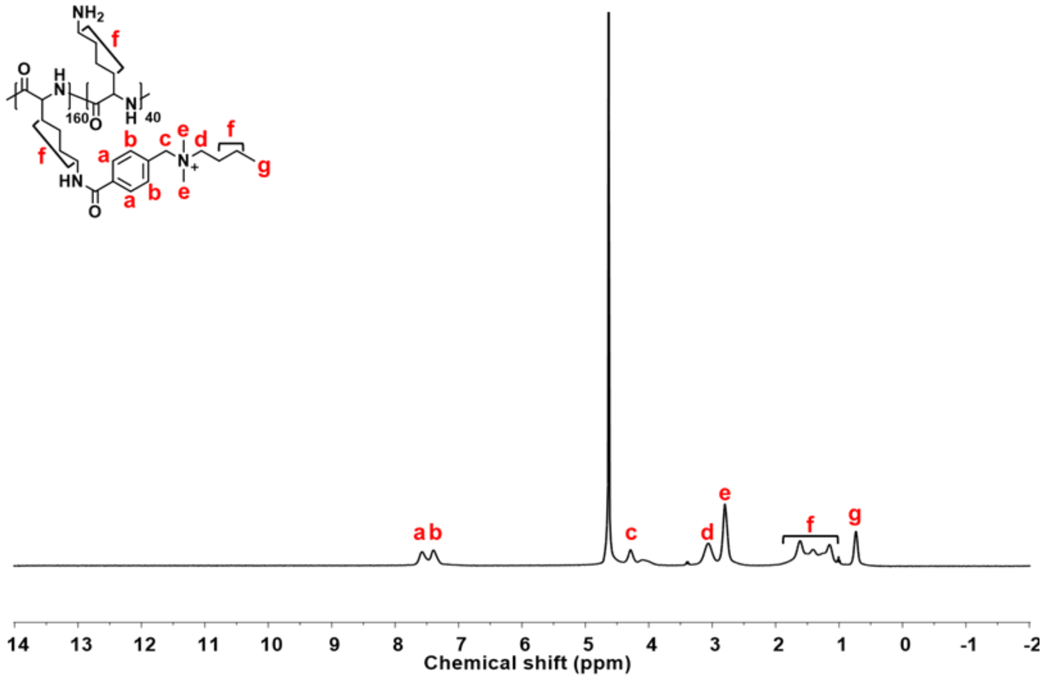

Figure S7. ^{1}H NMR of PDA-1 in D_2O

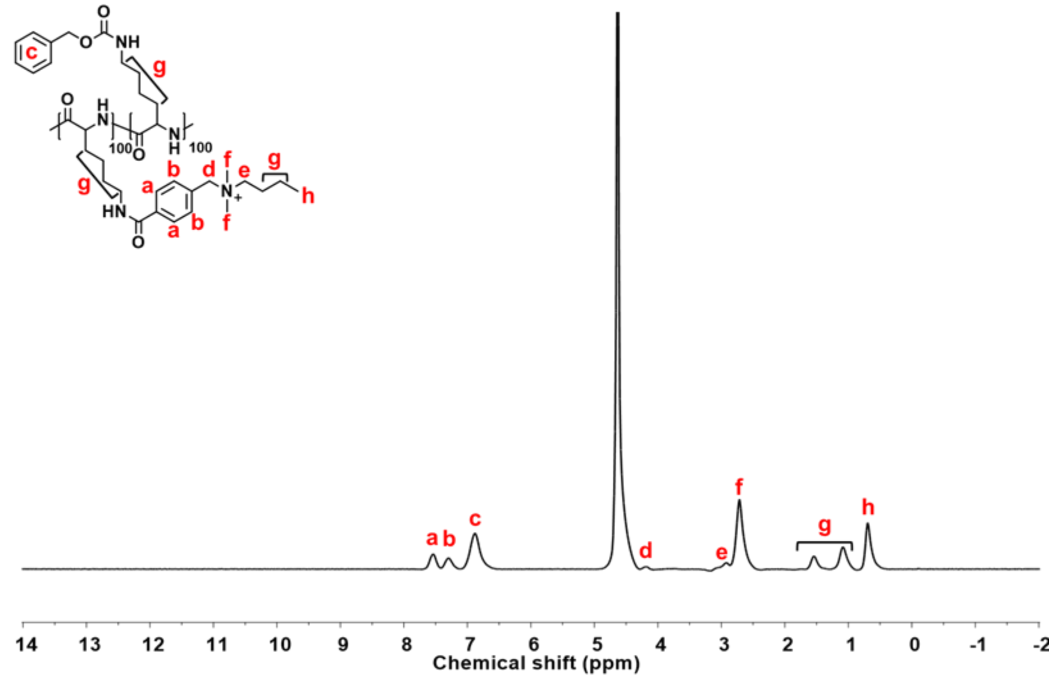

Figure S8. ^{1}H NMR of PDAC-2 in D_2O

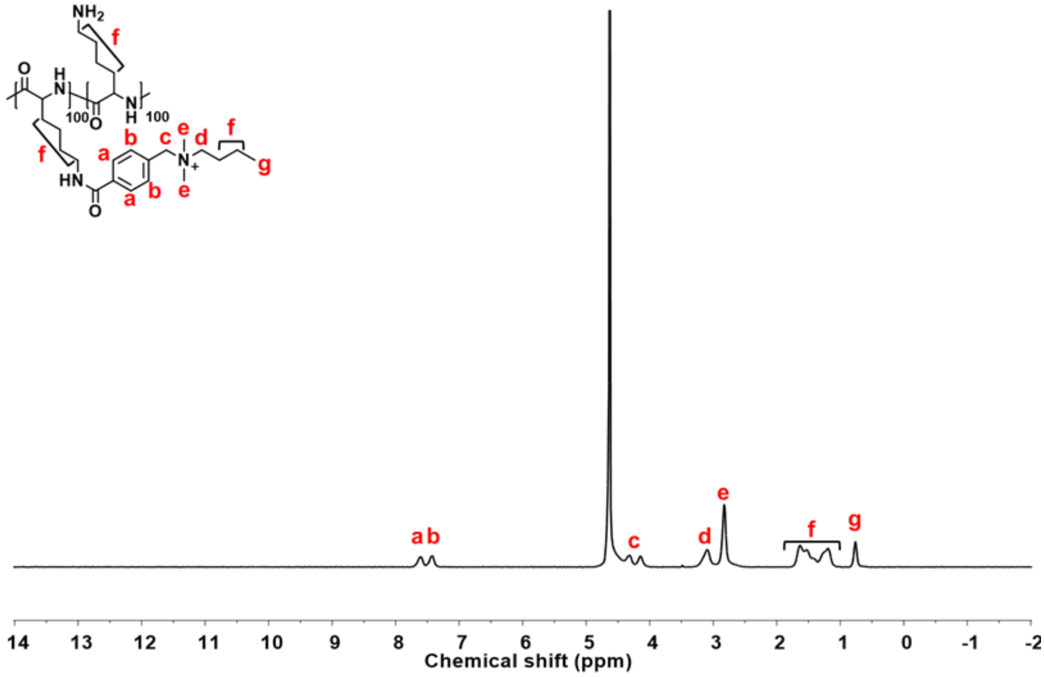

Figure S9. ^{1}H NMR of PDA-2 in D_2O

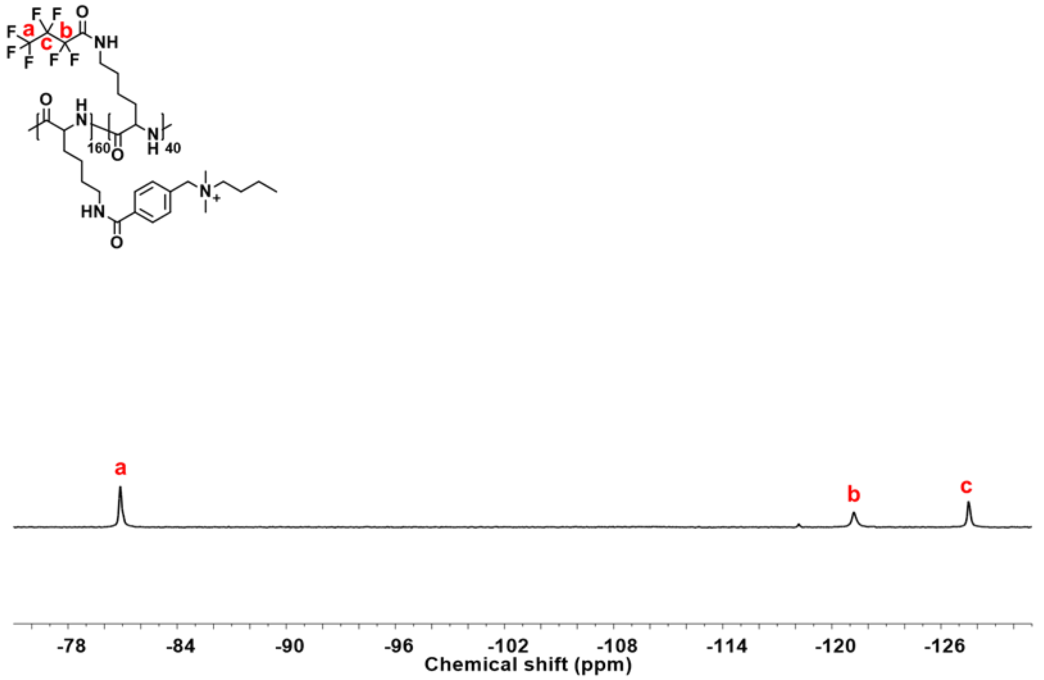

Figure S10. ^{19}F NMR of FHP-1 in D_2O

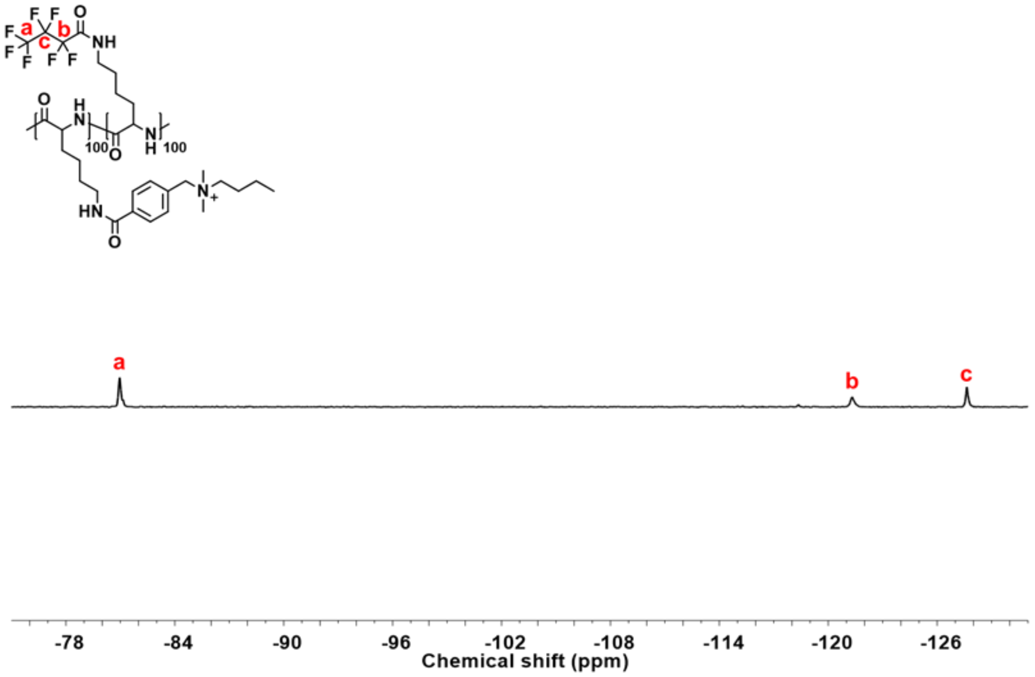

Figure S11. ^{19}F NMR of FHP-2 in D_2O

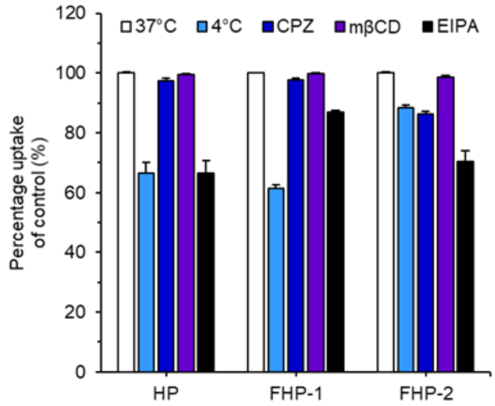

Figure S12. Cellular uptake mechanism of the fluorinated mitochondria-disrupting helical polypeptides. Cells were pre-treated with various endocytosis inhibitors such as CPZ, mβCD, and EIPA for 30 min. Subsequently, the cells were treated with polypeptides for 4 h, and the cellular uptake level was measured by flow cytometry.

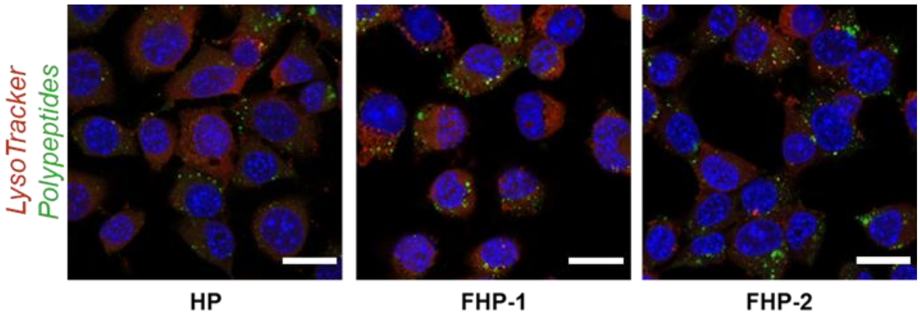

Figure S13. Endo/lysosomal escape of polypeptides. Cells were stained with LysoTrackerRed for 30 min. Subsequently, the stained cells were treated with FITC conjugated polypeptides for 3 h and endo/lysosomal escape of polypeptides were observed by CLSM. The scale bar represents 20 μm.

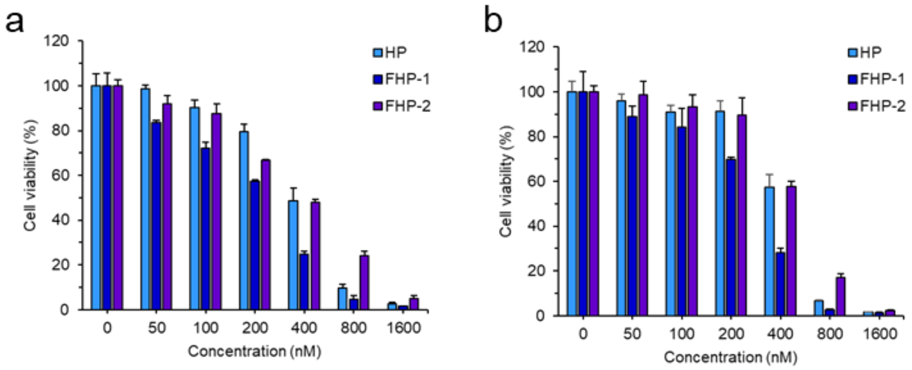

Figure S14. Cytotoxic effect of polypeptides against a) HCT116 (human colon carcinoma) and b) Lewis lung carcinoma (LLC). Relative cell viabilities were evaluated by MTT assay. Data are presented as mean ± S.D. (n=3).

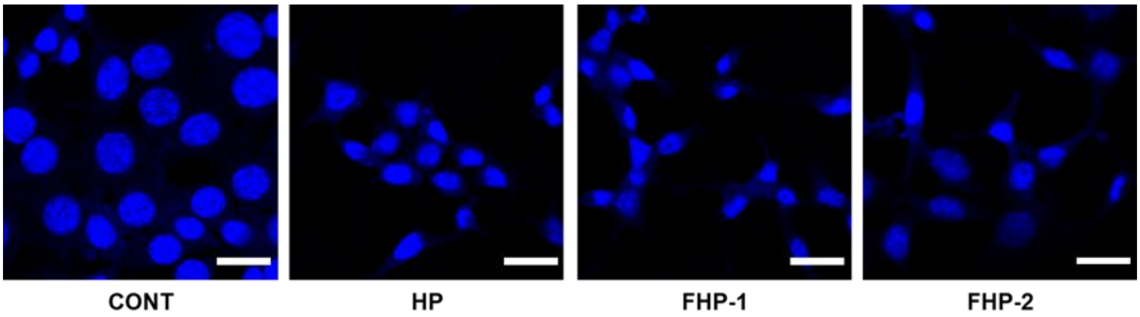

Figure S15. Nuclear condensation induced by the fluorinated mitochondria-disrupting helical polypeptides. Cells were treated with the polypeptides for 24 h and stained with DAPI to observe the nuclear morphological changes. The scale bar represents 20 μm.

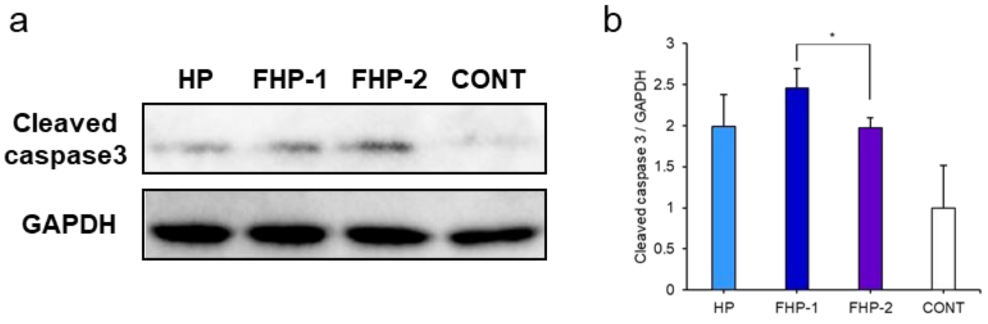

Figure S16. a) Western blotting of cleaved caspase 3 in CT26 cells. GAPDH was used as a loading control. b) Quantification of western blotting of cleaved caspase 3 in CT26 cells. Data are presented as mean ± S.D. (n=3). *P < 0.05, unpaired Student's t-test.

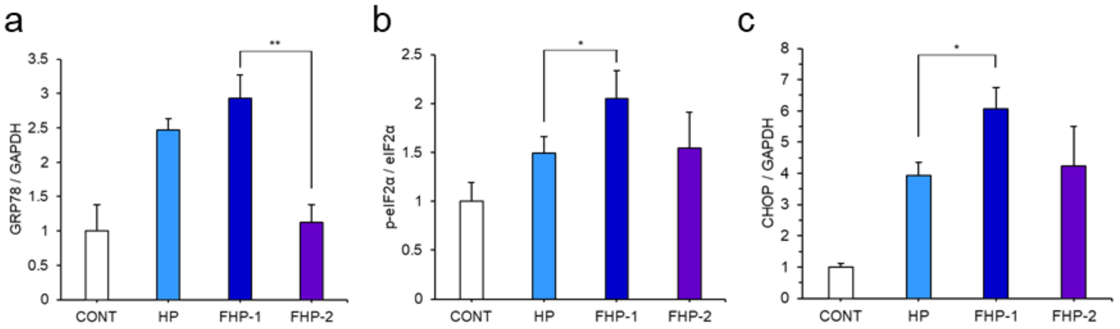

Figure S17. Quantification of ER stress related proteins western blotting in CT26 cells. Relative ratio between GRP78 and GAPDH (a), p-eIF2α and eIF2α (b), CHOP and GAPDH (c). Data are presented as mean ± S.D. (n=3). *$P < 0.05$, **$P < 0.01$, unpaired Student's t-test.

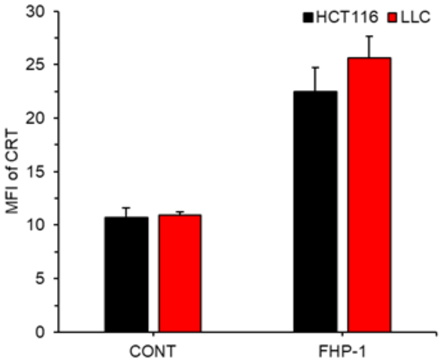

Figure S18. MFI of CRT in HCT116 and LLC cell lines after FHP-1 treatment for 24 h. Data are presented as mean ± S.D. (n=3).

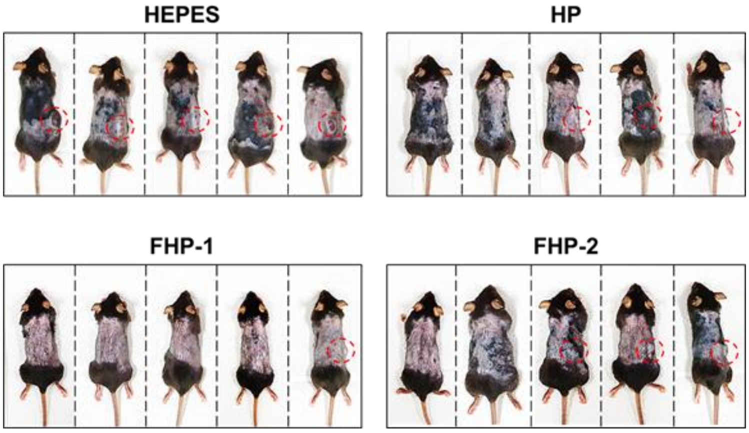

Figure S19. Representative images of vaccination experiment in LLC cells on Day 21.

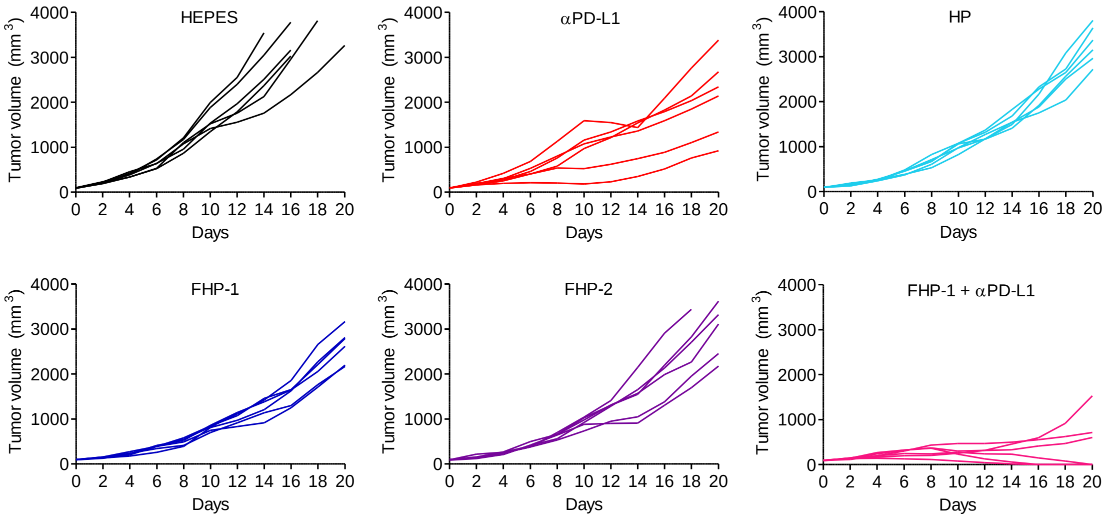

Figure S20. Individual tumor growth curves of CT26 bearing mice after treatment with polypeptide and/or αPD-L1. The tumor volume was measured every other day until the end of the study. Each line represents an individual mouse's tumor growth.

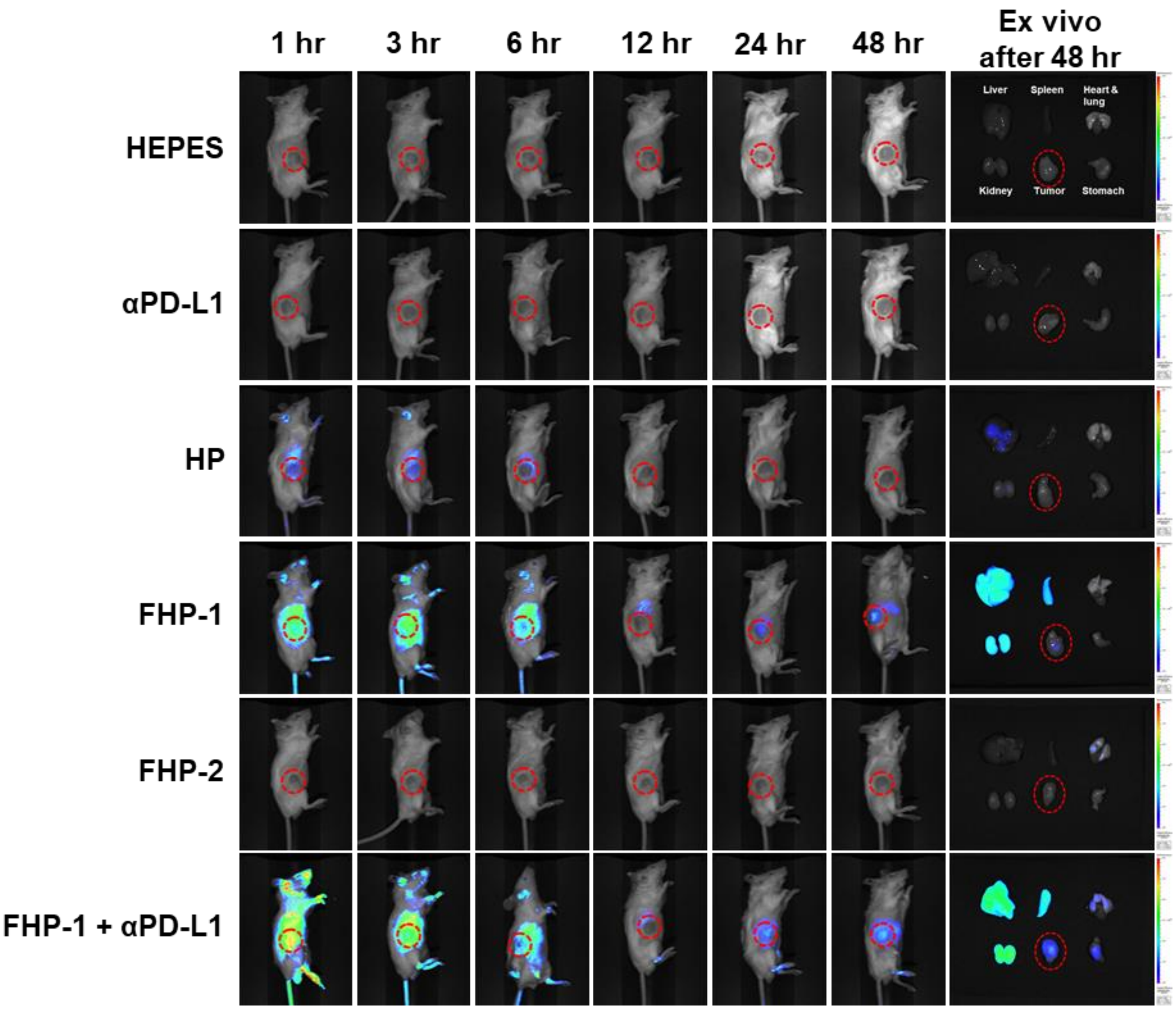

Figure S21. Biodistribution of the fluorinated mitochondria-disrupting helical polypeptides.

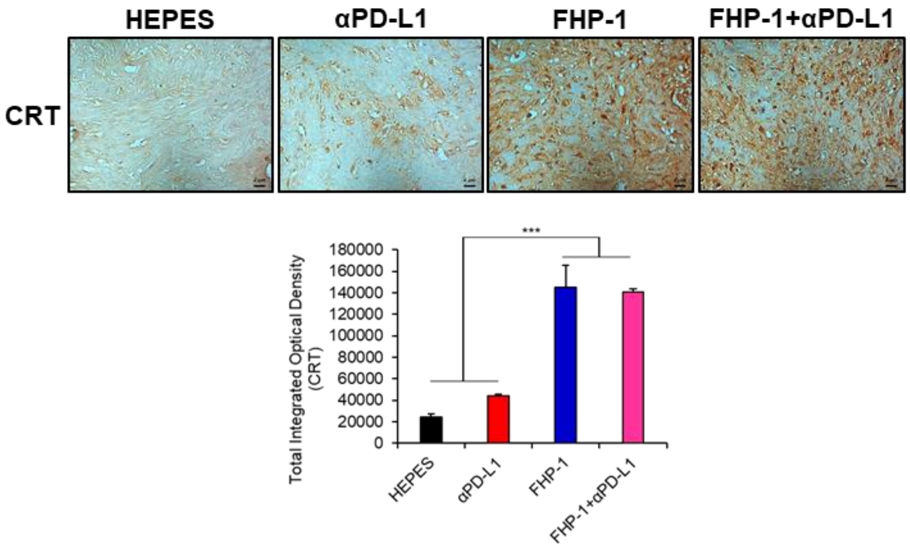

Figure S22. Immunohistochemistry images of CRT exposure in LLC tumor tissues. The quantitative data represented as mean ± S.D. (n=3); ****P* < 0.001, one-way ANOVA with Tukey's multiple comparisons test.

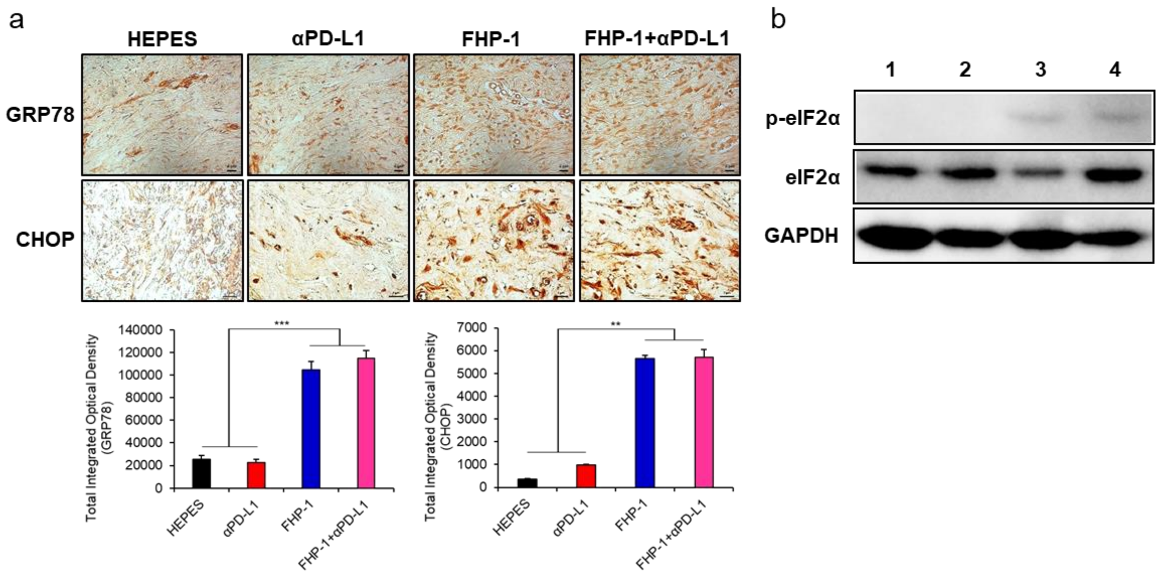

Figure S23. a) Immunohistochemistry images of GRP78 and CHOP in CT26 tumor tissues. The quantitative data represented as mean ± S.D. (n=3); ** $P < 0.01$, ***$P < 0.001$, one-way ANOVA with Tukey's multiple comparisons test. b) Western blotting of phosphorylation of eIF2α in tumor tissues (Lane 1: HEPES, Lane 2: αPD-L1, Lane 3: FHP-1, Lane 4: FHP-1+αPD-L1).

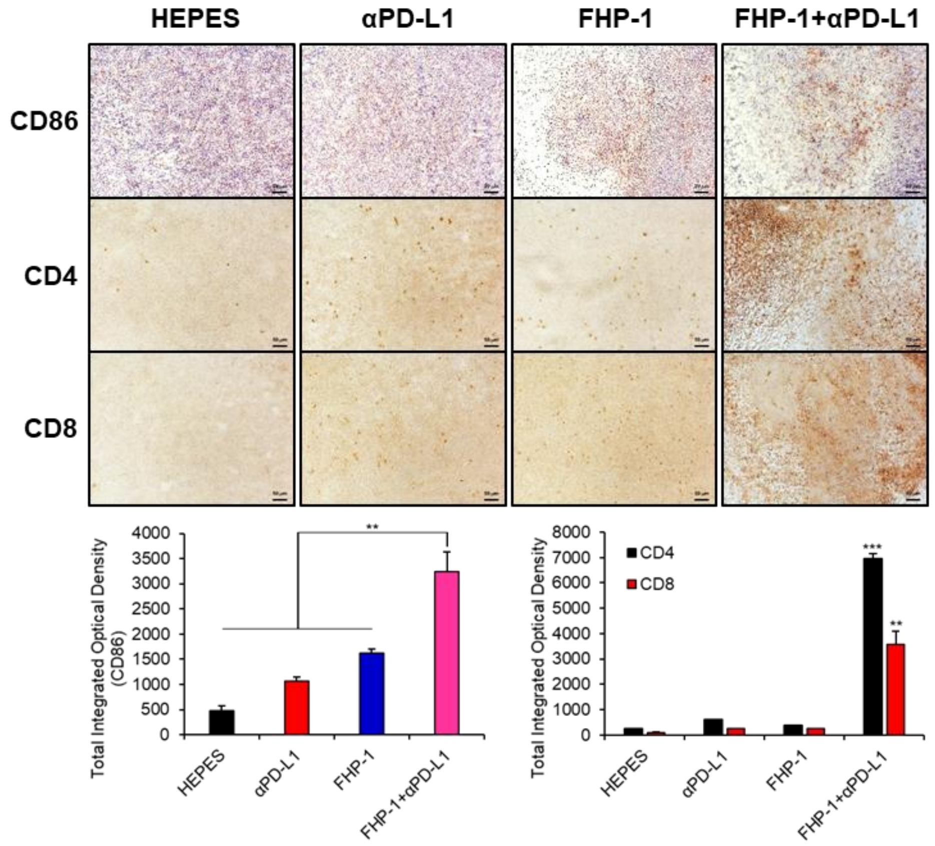

Figure S24. Tumor tissues were stained with CD86-, CD4-, and CD8-specific Ab. Original magnification: ×100. The scale bar represents 10 μm. The quantitative data represented as mean ± S.D. (n=3); ** $P < 0.01$, ***$P < 0.001$, one-way ANOVA with Tukey's multiple comparisons test.

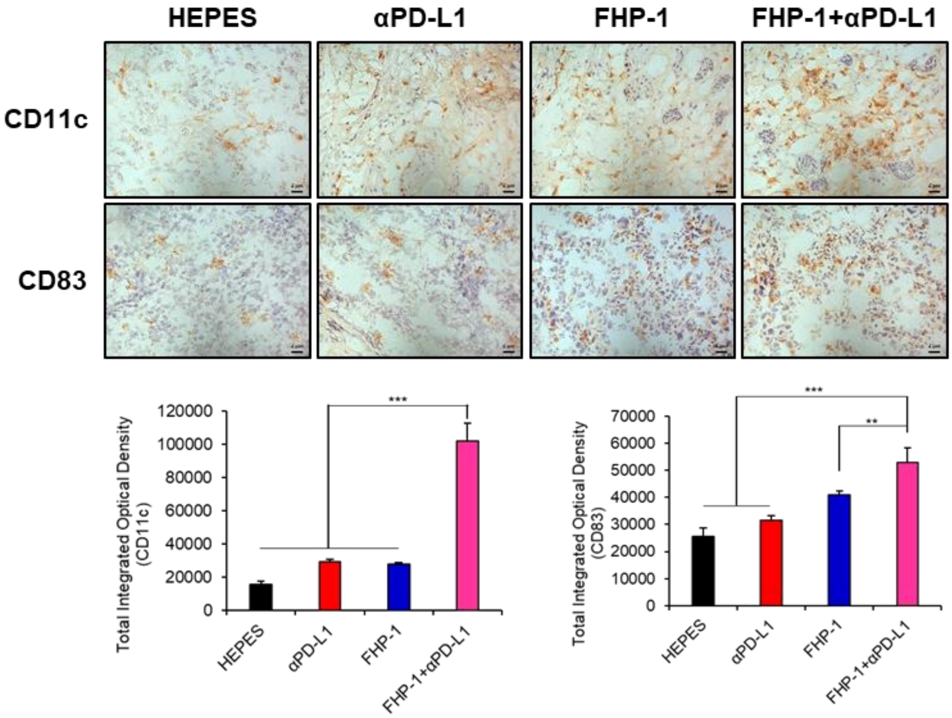

Figure S25. Immunohistochemistry images of CD11c and CD83 of tumor draining lymph node in LLC mouse model. The quantitative data represented as mean ± S.D. (n=3); ** $P < 0.01$, ***$P < 0.001$, one-way ANOVA with Tukey's multiple comparisons test.

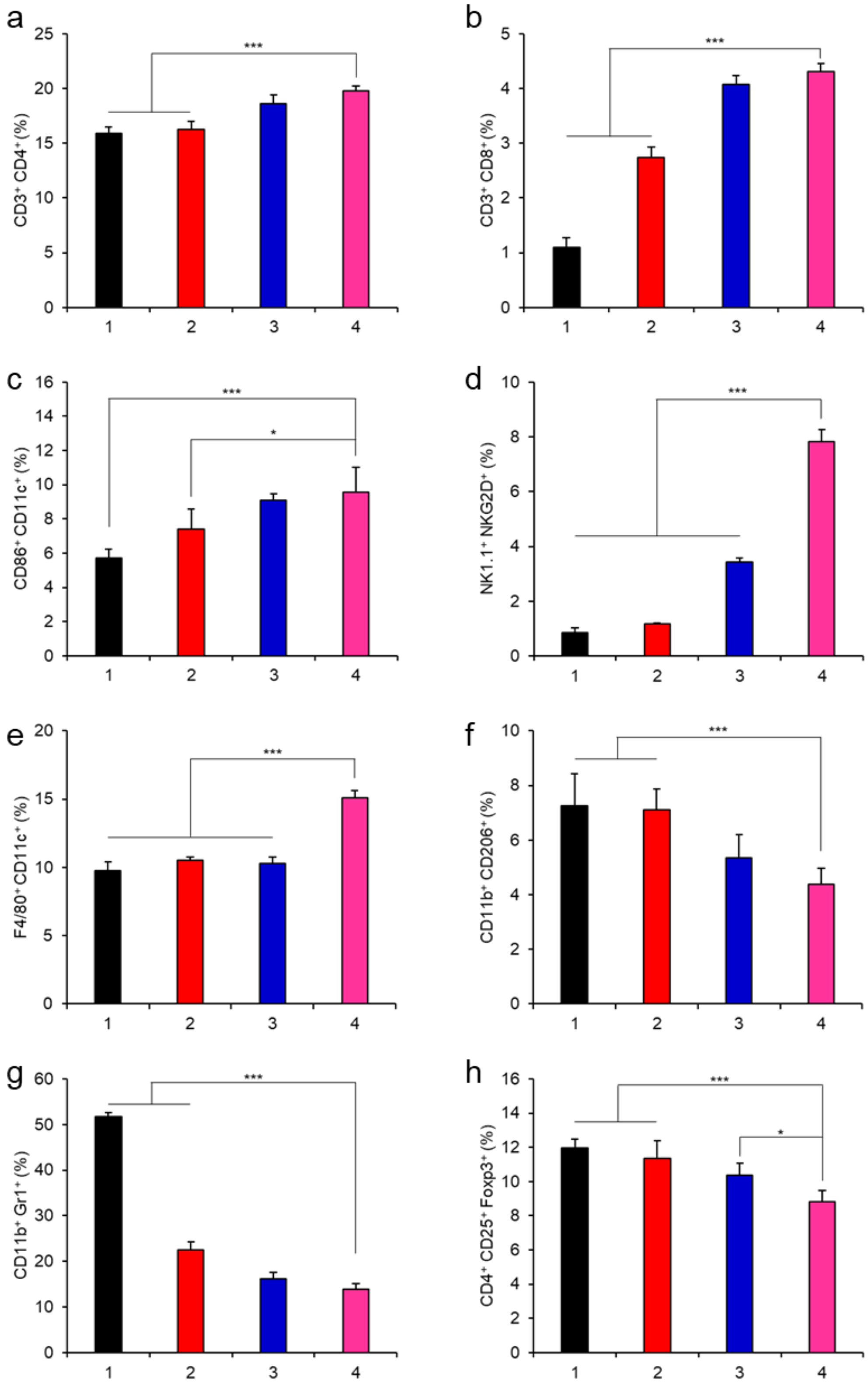

Figure S26. FACS analysis of tumor infiltrating immune cells in CT26 mouse model. $CD4^+$ T cells (a), $CD8^+$ T cells (b), DCs (c), NK cells (d), M1 macrophages (e), M2 macrophages (f), MDSCs (g), Tregs (h). (Group 1: HEPES, Group 2: αPD-L1, Group 3: FHP-1, Group 4: FHP-

1+ αPD-L1). Data are presented as mean ± S.D. (n=5). *P < 0.05, ***P < 0.001, one-way ANOVA with Tukey's multiple comparisons test.

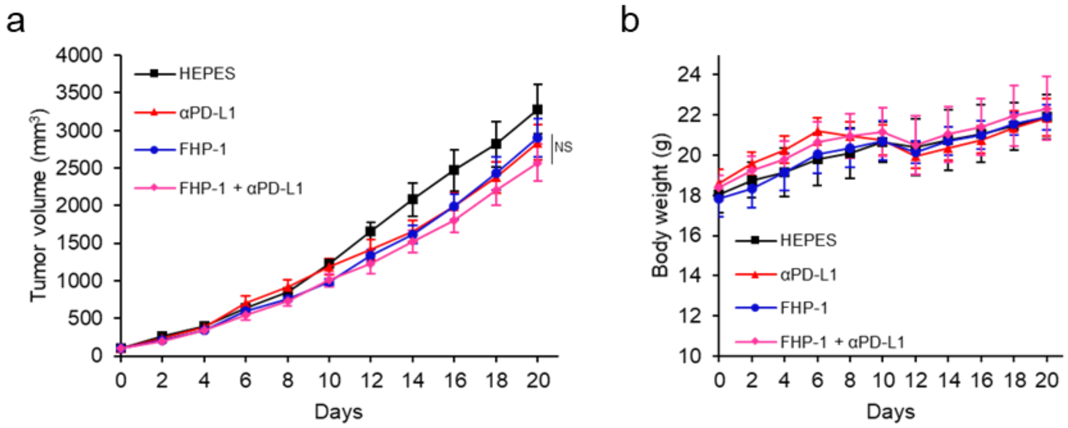

Figure S27. Tumor inhibition study in BALB/c nude mice. a) NCI-H460 cells were injected subcutaneously to BALB/c nude mice. When the average volume of established tumor reached 100 mm^3, the tumors were injected with FHP-1 and/or αPD-L1 following the schedule shown in Figure 5a. b) Body weight was measured every other day. Data represented as mean ± S.D. (n=6). NS: nonsignificant, one-way ANOVA with Tukey's multiple comparisons test.

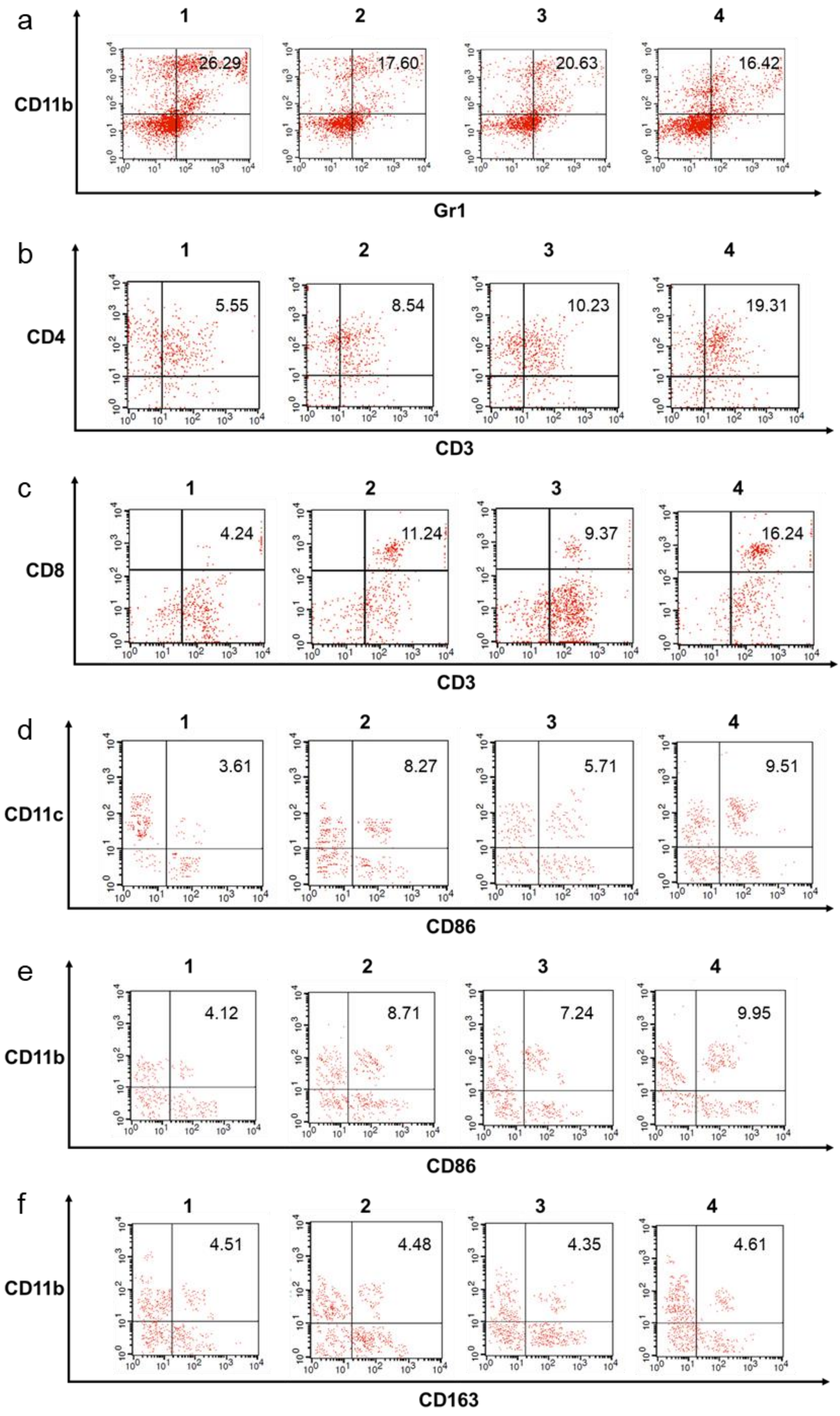
a
1
2
3
4
CD11b
26.29
17.60
20.63
16.42
Gr1
b
CD4
5.55
8.54
10.23
19.31
CD3
c
CD8
4.24
11.24
9.37
16.24
CD3
d
CD11c
3.61
8.27
5.71
9.51
CD86
e
CD11b
4.12
8.71
7.24
9.95
CD86
f
CD11b
4.51
4.48
4.35
4.61
CD163

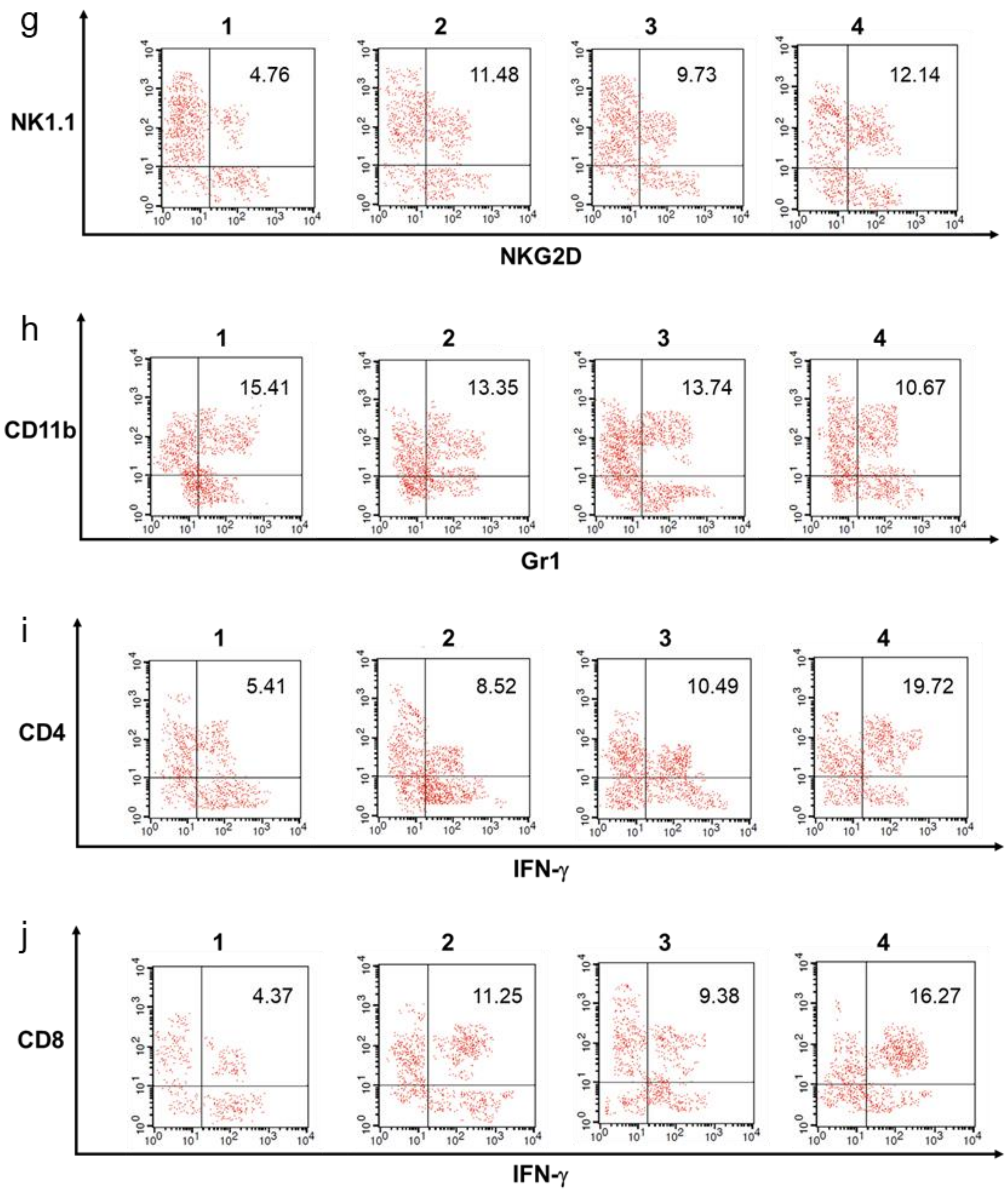

Figure S28. Representative images of flow cytometric analysis. a) MDSCs in spleen. b-j) FACS analysis of tumor infiltrating immune cells in LLC mouse model. $CD4^+$ T cells (b), $CD8^+$ T cells (c), DCs (d), M1 macrophages (e), M2 macrophages (f), NK cells (g), MDSCs (h), IFN-$\gamma^+$$CD4^+$ T cells (i), IFN-$\gamma^+$$CD8^+$ T cells (j). (Group 1: HEPES, Group 2: αPD-L1, Group 3: FHP-1, Group 4: FHP-1+αPD-L1).

	HEPES	αPD-L1	FHP-1	FHP-1 + αPD-L1
Bilirubin (mg dL^{-1})	≤ 0.1	< 0.1	< 0.1	≤ 0.1

Table S1. Measurement of bilirubin level in blood for liver toxicity.

Cell type	Ab
MDCS	PE-conjugated anti-mouse CD11b Ab (e-Bioscience)<br>PerCP-eFluor 710 conjugated anti-mouse Ly-6G (Gr-1) Ab (e-Bioscience)
Treg	PerCP-CY5.5-conjugated anti-CD4 Ab (BD Biosciences)<br>PE-conjugated anti-CD25 Ab (eBioscience)<br>APC-conjugated anti-Foxp3 Ab (eBioscience)
T cell	PerCP-CY5.5-conjugated anti-CD4 (BD Biosciences)<br>FITC rat anti-mouse CD3 (BD Biosciences)<br>PerCP-CY5.5 anti-mouse CD8a (BD Biosciences)
DC	FITC hamster anti-mouse CD11C (BD bioscience)<br>APC Rat anti-Mouse CD86 (BD Bioscience)
NK cell	APC anti-mouse NK1.1 (e-Bioscience)<br>ANTI-MOUSE CD314 (NKG2D) PE (e-Bioscience)
M1 macrophage	PE-anti-mouse CD11b Ab (BD Biosciences)<br>APC Rat anti-Mouse CD86 (BD Bioscience)<br>FITC hamster anti-mouse CD11c (BD Biosciences)<br>APC anti-mouse F4/80 (e-Bioscience)
M2 macrophage	PE-anti-mouse CD11b Ab (BD Biosciences)<br>PerCP-eFluor 710 anti-mouse CD163 (e-Bioscience)<br>FITC Rat Anti-CD11b (BD Biosciences)<br>Alexa Fluor® 647 Rat Anti-Mouse CD206 (BD Biosciences)

Table S2. The primary Abs used in FACS analysis of tumor infiltrating immune cells and splenocytes.
